# Complexity and ultrastructure of infectious extracellular vesicles from cells infected by non-enveloped virus

**DOI:** 10.1038/s41598-020-64531-1

**Published:** 2020-05-14

**Authors:** Jie E. Yang, Evan D. Rossignol, Deborah Chang, Joseph Zaia, Isaac Forrester, Kiran Raja, Holly Winbigler, Daniela Nicastro, William T. Jackson, Esther Bullitt

**Affiliations:** 1 Department of Physiology & Biophysics, Boston University School of Medicine, Boston, MA 02118 United States; 2 Department of Biochemistry, Boston University School of Medicine, Boston, MA 02118 United States; 3 Department of Biochemistry, Baylor College of Medicine, Houston, United States; 4 Department of Microbiology & Immunology, University of Maryland School of Medicine, Baltimore, MD 20201 United States; 5 Departments of Cell Biology and Biophysics, University of Texas Southwestern Medical Center, Dallas, TX 75235 United States; 6 Present Address: Department of Biochemistry, University of Wisconsin, Madison, WI 53706 United States; 7 Present Address: Ragon Institute of MGH, MIT, and Harvard, Cambridge, MA 02139 United States; 8 Present Address: Department of Biochemistry and Biophysics, University of Pennsylvania, Philadelphia, PA 19104 United States

**Keywords:** Mechanisms of disease, Electron microscopy, Infectious diseases, Viral infection, Infection, Biophysics, Cell biology, Structural biology, Diseases, Pathogenesis

## Abstract

Enteroviruses support cell-to-cell viral transmission prior to their canonical lytic spread of virus. Poliovirus (PV), a prototype for human pathogenic positive-sense RNA enteroviruses, and picornaviruses in general, transport multiple virions *en bloc* via infectious extracellular vesicles, 100~1000 nm in diameter, secreted from host cells. Using biochemical and biophysical methods we identify multiple components in secreted microvesicles, including mature PV virions; positive-sense genomic and negative-sense replicative, template viral RNA; essential viral replication proteins; and cellular proteins. Using cryo-electron tomography, we visualize the near-native three-dimensional architecture of secreted infectious microvesicles containing both virions and a unique morphological component that we describe as a mat-like structure. While the composition of these mat-like structures is not yet known, based on our biochemical data they are expected to be comprised of unencapsidated RNA and proteins. In addition to infectious microvesicles, CD9-positive exosomes released from PV-infected cells are also infectious and transport virions. Thus, our data show that, prior to cell lysis, non-enveloped viruses are secreted within infectious vesicles that also transport viral unencapsidated RNAs, viral and host proteins. Understanding the structure and function of these infectious particles helps elucidate the mechanism by which extracellular vesicles contribute to the spread of non-enveloped virus infection.

## Introduction

Enteroviruses are responsible for many widespread human diseases, including poliomyelitis (poliovirus; PV), hand-foot-and-mouth disease (Coxsackievirus; CV), and a recent respiratory infection outbreak in the United States of enterovirus D68^1^. PV has been intensively studied for over 60 years^2^ as a model system for studying infection by non-enveloped, positive-sense (+) single-stranded RNA viruses. In the typical life cycle, PV enters host cells, releases its (+) viral RNA (vRNA) from the capsid, and hijacks the host cell machinery to initiate viral protein translation. In combination, viral (e.g. viral proteins 2BC, 2 C, 3 AB, 3A) and host cell proteins induce intracellular membrane production and remodeling for construction of viral replication complexes, where template negative-sense (−) vRNA is generated^3,4^. These replication “factories” are essential for the massive generation of virions that then exit the cell to infect new hosts.

As important as replication, virion exit is a critical aspect for the spread of viral infection. Enveloped viruses are surrounded by a viral membrane, providing an elegant mechanism to enter and exit host cells through membrane fusion of the viral and host cell membranes, and budding from the host cell plasma membrane, respectively^5^. In contrast, the mechanism by which non-enveloped (naked) virions cross the cell membrane barrier is less well understood. The predominant exit strategy for non-enveloped viruses was thought to be host cell lysis, but there is now mounting evidence that non-enveloped virus exit is not as different from that of enveloped viruses as was previously thought^6^. Enteroviruses can exit cells non-lytically through “unconventional secretion” of extracellular vesicles (e.g.^7,8^). This alternative pathway provides cell-to-cell transmission of infection through *en bloc* virion transportation. Examples of non-enveloped viruses mediating non-lytic viral spread through secreted vesicles include hepatitis A virus (HAV), Coxsackievirus and PV^7–10^. These studies on non-lytic spread have demonstrated that vesicles isolated from the media of infected cells are sufficient to infect new cells. However, the structure, content and any additional roles of these extracellular vesicles in the spread of infection have not yet been well-characterized.

Two-dimensional transmission electron microscopy (TEM) of negatively stained samples has provided insights into morphological features of exosomes released from uninfected (e.g.^11^) and from virus-infected cells^7,9^. Despite such great advances, traditional staining introduces dehydration and distortion of the biological samples. The three-dimensional (3-D) structural features of native extracellular vesicles from virus-infected cells have not been defined, nor has there been a comparison between vesicles from infected and uninfected cells. Plunge-freezing allows near-native preservation of biological samples, which can then be imaged by cryo-electron microscopy (cryo-EM) for two-dimensional data, and by cryo-electron tomography (cryo-ET) to reveal 3-D reconstructions of pleiomorphic structures at nanometer to near-atomic resolution (e.g.^12,13^).

Biochemically and structurally, we analyzed secreted vesicles from PV-infected cells for viral RNAs and proteins, and visualized their 3-D ultrastructure by cryo-ET, showing that vesicles secreted by PV-infected cells contain infectious viruses and a diverse set of proteins and viral RNAs.

## Results

**Figure 1:**
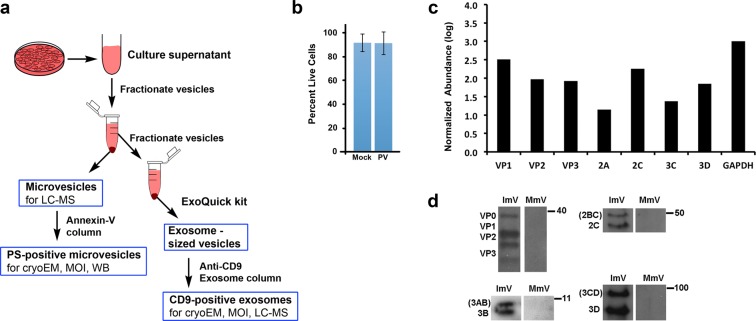
Sample preparation and the presence of PV proteins in secreted vesicles. (**a**) Schematic of collection and purification of microvesicles and exosomes. Annexin-V-coated magnetic beads were used to purify microvesicles that include phosphatidylserine (PS) in their outer membrane. (**b**) Cell viability at the time of vesicle collection, 8 hpi, showing minimal cell death. (**c**) Mass spectrometric analysis of poliovirus protein abundance in isolated infectious microvesicles collected at 8 hpi. Protein abundance was normalized to the GAPDH level of PV-infected cells, multiplied by 1000 and presented as log_10_ (Normalized Abundance). (**d**) Viral structural proteins (VP0, VP1, VP2, VP3) and non-structural proteins (2C, 2BC 3D, 3CD, 3A, 3AB) were identified in PS-containing infectious microvesicles (ImVs), detected via western blot using polyclonal antibodies against 2C, 3A, 3D, and/or GAPDH, from samples taken at 5 hpi (anti-3A) or 8 hpi. For each antibody used, the ImV and mock-infected microvesicles (MmV) lanes were run on the same gel, and the same vertical position on the gel is shown for both lanes. Full western blots from which the lanes were taken are all shown in Supplementary Fig. S2: VP proteins, Suppl Fig. S2a; 2C protein, Suppl Fig. S2b; 3D protein, Suppl Fig. S2c; 3A protein, Suppl. Figure S2d.

Extracellular vesicles serve multiple roles for cells, including cell signaling and transport of functional proteins, coding RNAs, and/or non-coding RNAs^14–16^. Vesicles with a diameter of 100–1000 nm secreted from PV-infected cells were shown to carry PV virions^10^. Therefore, we analyzed extracellular vesicles isolated by size using the well-established fractionation method of differential centrifugation for microvesicles (100–1000 nm)^17^. Phosphatidylserine (PS) -containing microvesicles were purified for additional analysis, as diagrammed in Fig. 1a (see also Materials & Methods).

To follow PV-infection of HeLa cells and determine efficiency of viral infection, control (mock-infected) and PV-infected cells were harvested at 3 and 7 hours post infection (hpi) for control cells, and 3, 4, 5, 6, and 7 hpi for PV-infected cells. Cytoplasmic lysates were then probed for viral proteins. The non-structural viral protein 3CD was produced by PV-infected HeLa cells at 3 hpi, and its levels stayed constant from 3 to 7 hpi; production of the viral polymerase 3Dpol started by 3 hpi and reached a high level at 4 hpi (Supplementary Fig. S1a), consistent with the literature (e.g.^18^).

### Capsid proteins and essential viral replication proteins were present within extracellular vesicles secreted from PV-infected cells

To characterize secreted vesicles and their contents, we collected and size-fractionated secreted vesicles from infected and mock-infected cells (Fig. 1a). Because the release of PV-containing infectious microvesicles (ImVs) peaks at approximately 8 hpi^10^, at which time infected cells have not yet begun to lyse^8,10^, vesicles were collected at 8 hpi. To rule out the presence of significant contaminants from intracellular contents in this study, cell viability greater than 90% was confirmed for both mock- and virus-infected cells (Fig. 1b). Samples included all vesicles secreted after the cells were washed and non-FBS-containing media was added at 4 hpi, the time of peak replication. Liquid chromatography-mass spectrometry (LC-MS) of 100–1000 nm diameter vesicles (called here ‘microvesicles’) secreted from infected cells resulted in the detection of the non-capsid, essential viral replication 2A-, 2C-, 3C- and 3D- containing proteins, in addition to viral capsid proteins VP1-VP3 (Fig. 1c). Consistent with the LC-MS data from size-fractionated microvesicles, western blot analyses (Fig. 1d; full western blots for all samples are shown in Supplementary Fig. S2) further confirmed the presence of non-capsid viral proteins in microvesicles that contained the membrane phospholipid PS, a sub-population of microvesicles that had been shown to be infectious^10^. Specifically, the PV proteins 2BC, 2 C, 3 A, 3 AB, 3CD, and 3D were identified in PS-containing microvesicles secreted from PV-infected cells but not in those secreted from mock-infected cells (Fig. 1c,d). We did not see 2BC, 3 AB, and 3A in the LC-MS data, likely due to the loss of membrane-associated proteins 2B and 3A in the LC-MS sample preparation.

### Replicative and genomic RNA were present in secreted infectious microvesicles

**Figure 2:**
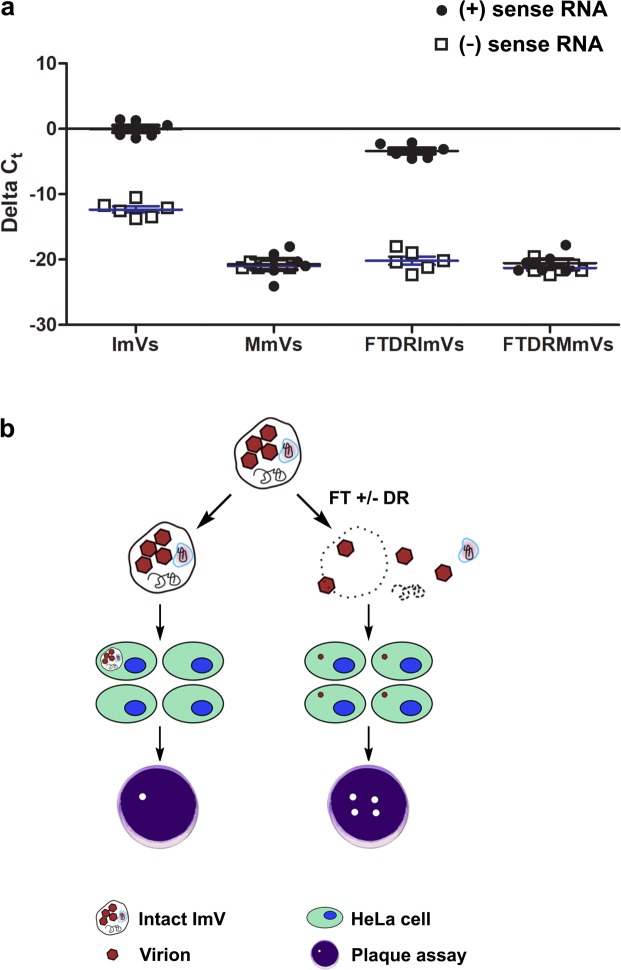
Infectious microvesicles (ImV) carry viral RNAs. (**a**) RT-qPCR data quantifying both (+) and (−) sense viral RNA (vRNA) from infectious microvesicles (ImV) and mock microvesicles (MmV) that were collected at 8 hpi (see Methods for details). vRNA was measured after extracellular vesicles underwent: 1) no treatment (labeled ImVs or MmVs), or 2) freeze-thaw & detergent (1% sodium deoxycholate) & RNase treatment to break open vesicles and degrade unencapsidated RNAs (labeled DRImV for infectious or DRMmV for mock-infected samples). RT-qPCR relative quantification was calculated as ΔC_t_ where ΔC_t_ = (C_t_ of endogenous control gene (GAPDH)) – (C_t_ of gene of interest (vRNA)), using GAPDH of whole cells for normalization. (**b**) Schematic of the experimental design for determining the infectivity of untreated infectious microvesicles (ImVs) as compared to infection by ImVs treated by either freeze/thaw alone (FT) or FT, detergent, and RNase (DR). Serial dilutions of sample were used in plaque assays to quantify the number of infectious sites by visual inspection for cell death.

The initiation of PV replication for packaging (+) vRNA within virions requires the presence of template, replicative (−) vRNA. Therefore, we examined the content of PS-positive infectious microvesicles with regard to the presence of vRNA by RT-PCR (Materials & Methods). No amplicons were detected above the threshold set at a fixed signal intensity (0.475) for all experiments. To confirm the absence of DNA contamination from the total RNA extraction/purification process, SuperScript III Reverse Transcriptase was omitted from the “master mix” for reverse transcription, and no amplification occurred in total RNA from PV-infected cells, when either positive-sense (genomic) or negative-sense (anti-genomic, replicative) viral RNA was used as the probe (Supplementary Fig. S3b,c). To test for nonspecific binding introduced by primer dimers and secondary structures of the primers, RT-qPCR was performed on RNase/DNase-free water including primers either against positive- or negative-sense RNA (Supplementary Fig. S3d,e). In contrast, significant amplifications corresponding to either (+) or (-) vRNA occurred in PV-infected cells when the proper primers, enzymes, and reaction agents were present (Supplementary Fig. S3f,g). In addition to these system controls, we analyzed melt curves of PV-infected samples to test whether the dye qPCR assay (SYBER) produced single, specific products. The appearance of single peaks indicated one melting event, corresponding to the positive target amplicon (Supplementary Fig. S3h) or the negative target (Supplementary Fig. S3i). No amplification was observed in mock-infected cells (Supplementary Fig. S3j). Consistently, both (+) vRNA and (-) vRNA were detected in PV-infected cells, and were absent in mock-infected cells (Supplementary Fig. S3k). We found significant decreases (*p* ≤ 0.001) in the raw/unnormalized cycle threshold, C_t_ (number of PCR cycles for the signal to exceed background) for PV ( + ) vRNA (genomic vRNA that can be used for both translation and as a template for (-) RNA synthesis) and for (-) vRNA (anti-genomic vRNA) within infectious microvesicles (ImVs) when compared to non-specific amplification from mock-infected microvesicles (MmVs) (Fig. 2a, ImVs *vs*. MmVs). These results established the presence of both (+) and (−) vRNA in ImVs.

The vRNA within secreted vesicles had been expected to be entirely virion-encapsidated vRNA because virion assembly is tightly regulated to encapsidate only (+) vRNA^19^. We therefore tested whether the newly identified intravesicular (−) vRNA was “free”, or was packaged within virion capsids that were inside infectious microvesicles. We exposed microvesicles prepared as diagrammed in Fig. 1a to a series of further treatments (Fig. 2b) that included membrane disruption to release the vesicle content by freeze-thaw and detergent (sodium deoxycholate), followed by RNase treatment to remove any unprotected (intravesicular yet unpackaged in capsids) RNA. Because membranes are disrupted, and capsids are resistant to and unaffected by freeze-thaw, detergent, or RNases (Supplementary Fig. S4), consistent with the literature^20–22^, the majority of (+) vRNA was still present in post-treated infectious microvesicles, as expected, whereas (−) vRNA was almost undetectable (C_t_ ≥ 37, a value determined based on the negative reverse transcription and non-template controls) (Fig. 2a). Interestingly, however, there was a small but significant decrease (Fig. 2a, ImVs *vs*. FTDR ImVs, *p* ≤ 0.05) in the abundance of (+) vRNA in post-treated infectious microvesicles. These data indicate that not all (+) vRNA was protected inside assembled virions, as capsids are well known to protect their internal RNA from RNase-mediated degradation^19,22^. The status of this non-encapsidated RNA, whether single- or double- stranded, is not known, as under the conditions of the experiments both forms would be digested by RNaseA.

### Host cell proteins identified by mass spectrometry analysis

Our data showed that less than 10% of the LC-MS identified protein peptides in microvesicles from PV-infected cells were viral proteins. Studying the components in more detail, we observed that the host cell protein components in microvesicles from mock-infected cells exhibited a much smaller diversity of proteins than infectious microvesicles from PV-infected cells (Supplementary Table S1). We identified a total of 65 host cell protein matches that were present in both independent experiments on infectious microvesicles, with three technical replicates combined per experiment, and a threshold cutoff of 0.999 probability. All five proteins identified in mock-infected sample (MmVs) matched proteins identified in ImVs (8% of the ImV proteins).

We then categorized functionally-related proteins using Ingenuity Pathway Analysis (IPA; Qiagen) software^23^. The significant pathways (-log p > 4), as calculated by the p-value of overlap (see Materials and Methods), are shown in Table 1. Five enzymes from the glycolytic pathway (-log p = 8.3) were identified in infectious microvesicles (ENO1 {ENOA}, TPI {TPIS}, PKM {KPYM}, ALDOA, GAPDH); names in braces are synonyms), as were eight proteins from the RhoGDI (Rho GDP dissociation inhibitor) pathway (-log p = 7.7) (ITGB1 {CD29}, GNAI3, CFL1, ACTB, EZR, RHOA, CD44, MSN). Seven proteins identified in infectious microvesicles are involved in caveolar-mediated and/or clathrin-mediated endocytic virus entry pathways (-log p = 4.4 or 4.9, respectively). The proteins include, for caveolar-mediated endocytosis, the enterovirus 70 protein receptor (CD55, also known as the CV A21 host-entry receptor DAF) and the echovirus receptor Integrin beta 1 (ITGB1) proteins^24–26^. Used for entry through clathrin-coated endocytosis by the enveloped arenavirus^27^, the host receptor TfR1 (TFRC) protein was also identified in infectious microvesicles secreted from PV-infected cells. Another pathway identified with high probability (-log p = 5.8) was actin cytoskeleton reorganization, including RAC proteins (Rho family GTPases), the tyrosine-protein kinase FYN, and additional actin cytoskeleton-regulating proteins (ITGB1, CFL1, RHOA, EZR, MSN).

**Table 1:** Canonical Pathways Identified in Microvesicle Samples.

Ingenuity Canonical Pathways	-log(p-value)	Ratio	Secreted infectious microvesicle (ImV) sample from PV-infected cells
			Identified Molecules
Glycolysis I	8.33	0.208	ALDOA,ENO1,GAPDH,PKM,TPI1
RhoGDI Signaling	7.65	0.0452	ACTB,ACTG1,CD44,CFL1,EZR,ITGB1,MSN,RHOA
Virus Entry via Endocytic Pathways	6.37	0.0556	ACTB,ACTG1,CD55,FOLR1,ITGB1,TFRC
Leukocyte Extravasation Signaling	6.08	0.0361	ACTB,ACTG1,CD44,EZR,ITGB1,MSN,RHOA
RhoA Signaling	6.08	0.0496	ACTB,ACTG1,CFL1,EZR,MSN,RHOA
Actin Cytoskeleton Signaling	5.8	0.0327	ACTB,ACTG1,CFL1,EZR,ITGB1,MSN,RHOA
Glucocorticoid Receptor Signaling	5.55	0.024	ACTB,ANXA1,HSPA8,KRT1,KRT10,KRT14,KRT2,KRT9
Signaling by Rho Family GTPases	5.46	0.029	ACTB,ACTG1,CFL1,EZR,ITGB1,MSN,RHOA
Mechanisms of Viral Exit from Host Cells	5.36	0.0976	ACTB,ACTG1,CHMP4B,PDCD6IP
Germ Cell-Sertoli Cell Junction Signaling	5.26	0.0359	ACTB,ACTG1,CFL1,ITGB1,RHOA,TUBB
Clathrin-mediated Endocytosis Signaling	4.9	0.0311	ACTB,ACTG1,HSPA8,ITGB1,TFRC,UBA52
Gluconeogenesis I	4.4	0.12	ALDOA,ENO1,GAPDH
Caveolar-mediated Endocytosis Signaling	4.36	0.0548	ACTB,ACTG1,CD55,ITGB1
Pyruvate Fermentation to Lactate	4.15	0.4	LDHA,LDHB
Regulation of Actin-based Motility by Rho	4.04	0.0455	ACTB,CFL1,ITGB1,RHOA
**Ingenuity Canonical Pathways**	**-log(p-value)**	**Ratio**	**Secreted mock microvesicle (MmV) sample from mock-infected cells (control)**
			**Identified Molecules**
Glucocorticoid Receptor Signaling	6.44	0.012	ACTB,KRT1,KRT10,KRT2
Mechanisms of Viral Exit from Host Cells	4.4	0.0488	ACTB,ACTG1
MSP-RON Signaling Pathway	4.08	0.0339	ACTB,ACTG1

A subset of the proteins detected by mass spectrometry is shown, selected based on analysis that identifies significantly represented cellular pathways. Cellular pathways were identified by the likelihood, p, that the presence of detected proteins is not due to random chance, as measured by -log (p)>4 (see Methods for further details). ‘Ratio’ is the number of proteins from that pathway that were identified in the samples, divided by the total number of pathway proteins. Only proteins identified in both experimental repeats were included in the analysis.

Although exosomes are typically smaller sized vesicles, some overlap between microvesicles and exosomes can be expected from a differential centrifugation separation^28,29^. Therefore, it is not surprising that components of the exosomal pathway were also identified in our size-fractionated infectious microvesicles. These components include PDCD6IP (programmed cell death 6 interacting protein, also known as ALIX), which participates in ESCRT-III recruitment^30^, syndecan binding protein (SDCBP), which is involved in the biogenesis and cargo loading of exosomes^31^, and several classical exosomal markers such as the ESCRT-III associated factor, increased sodium tolerance 1 (IST1)^32^ and CD9^33^ (Table S1).

The detection of these exosomal components led us to question whether PV infection might also exploit smaller exosome-like vesicles to transport virions and viral proteins from cell to cell, as has been shown for exosome-like virion-containing vesicles from HAV-infected cells^7^. To investigate their role in PV spread, exosomes shed from mock- and PV-infected cells were collected, using established size-based fractionation of 40–100 nm diameter vesicles and further purified using antibodies against the exosomal marker CD9 (see Materials and Methods), to avoid contamination of the exosome fraction with similarly sized free virions (28 nm diameter) and microvesicles, and used for functional infectivity characterization (plaque assays) and cryo-ET.

### Intact extracellular vesicles were infectious and the carried contents altered cellular conditions

**Figure 3:**
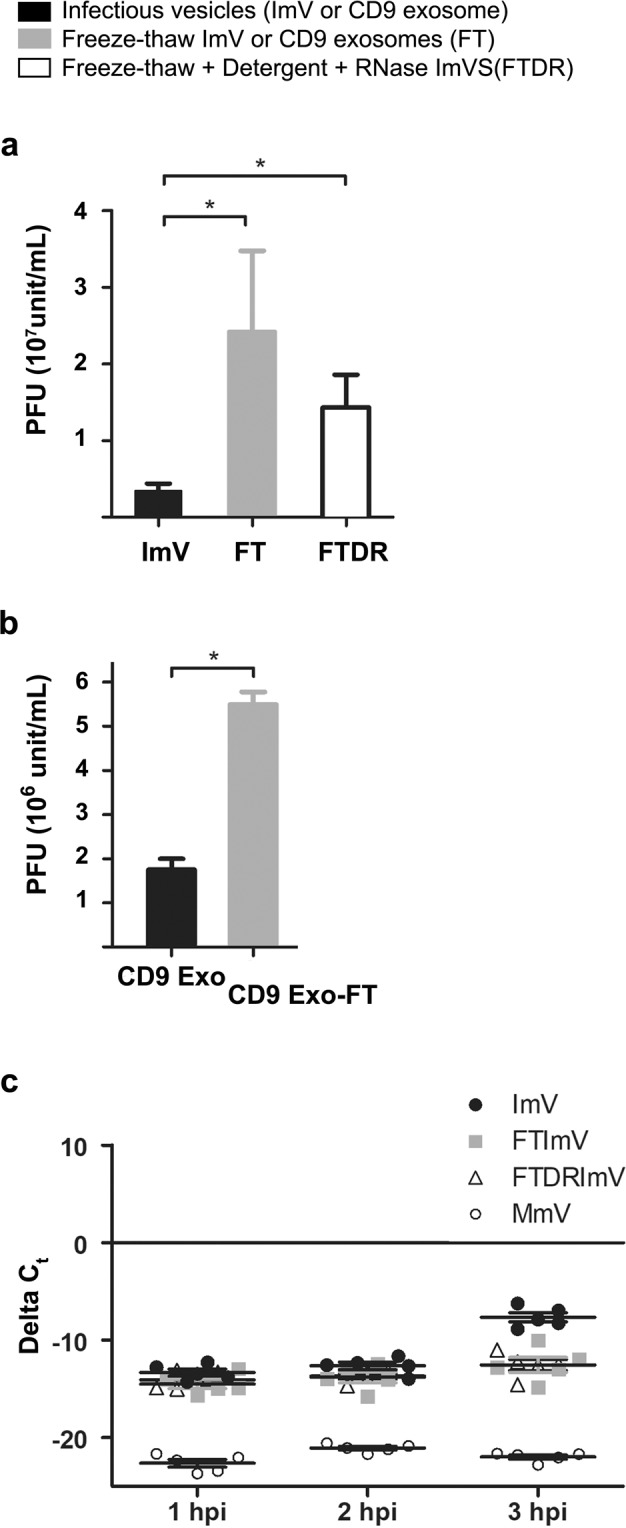
Extracellular vesicles from PV-infected cells are infectious. Infectivity of purified (**a**) infectious microvesicles (ImVs) and (**b**) CD9-positive exosomes (CD9 Exo) was quantified by standard plaque assay for virus titer, expressed as plaque-forming units (PFU) per mL; *p < 0.05. Samples were untreated, or freeze/thawed (FT), or freeze/thawed & detergent (1% sodium deoxycholate) & RNase treated (FTDR). Data are means ± standard deviations from at least three independent experiments. (**c**) Changes in viral RNA production from ImVs were measured by RT-qPCR. Relative quantification was calculated as ΔC_t_ where ΔC_t_ = (C_t_ of endogenous control gene (GAPDH)) – (C_t_ of gene of interest (vRNA)) from at least three independent experiments, using GAPDH of whole cells for normalization. Cells were infected with a constant number of virions, either transported in an intact extracellular vesicle, or as free virions after release from the vesicles, at a multiplicity of infection of 1 PFU per cell.

Previous studies demonstrated that microvesicles transport virions. Classic infectivity plaque assays showed that intact infectious microvesicles produced fewer infectious centers than intravesicular virions released from these vesicles by freeze/thaw^10^. Confirming these previous results, our quantification of plaque assays revealed a 10-fold increase in plaque-forming units (pfu) after disruption of the infectious microvesicle membrane and release of intravesicular virions by freeze/thaw (FT) prior to infection (Figs. 2b, 3a, ImV *vs*. FT ImVs). We also saw a greater than 3-fold increase in pfu for infection with freeze/thawed-detergent/RNase-treated infectious microvesicles as compared to intact infectious microvesicles. We note that the number of infectious centers (pfu) was not significantly different when cells were infected with intravesicular PV virions released by either method: freeze/thaw or freeze/thaw/detergent/RNase (FTDR) (Fig. 3a, FT *vs*. FTDR). Analogous experiments were completed on CD9-positive exosomes purified from the media of PV-infected cells. Exosomes from PV-infected cells were shown to be infectious, exhibiting a three-fold increase in plaque formation after disruption of the vesicles’ membranous structures by freeze/thaw (Fig. 3b).

A linear increase in the MOI by PV is known to result in a proportional increase in (+) vRNA production in infected cells^34^. This suggests that if the additional vesicle contents here identified by LC-MS have no effect on viral replication, the (+) vRNA produced by intact vesicles or by the virions released from broken vesicles would be expected to be comparable. For example, suppose one infectious vesicle that contains 10 virions is added onto 10 cells, either as an intact vesicle or first freeze/thawed to release the intravesicular virions. Whether 1 cell is infected with 10 virions (local MOI = 10 in one cell, and 9 cells are uninfected) by this intact infectious vesicle, or whether 10 cells are each infected with 1 virion released from the broken infectious vesicle (local MOI = 1 in one cell, 10 infected cells in total), the measured vRNA production would be the same. A larger increase in (+) vRNA production by intact infectious vesicles would suggest the presence of additional virulence factors provided within the vesicle.

Thus, to evaluate possible function(s) of the complex content of infectious vesicles during early-stage infection, we performed experiments to test whether intact vesicles resulted in an increase in (+) vRNA production in newly infected cells beyond equal viral replication for infection with treated (broken) or untreated (intact) vesicles. The total collected infectious microvesicles were divided into three equal aliquots and used to infect the cells with 1) untreated, intact infectious microvesicles, 2) freeze/thawed, broken microvesicles to release free virions, or 3) freeze/thawed, broken microvesicles that were subsequently subjected to detergent and RNase treatment. A time course of (+) vRNA production by host cells was measured using RT-qPCR. At an early infection stage (3 hpi), intact infectious microvesicles initiated a more rapid onset of viral replication, evidenced by an over 2-log increase in (+) vRNA production in host cells compared to both freeze/thawed or freeze/thawed-detergent/RNase treated groups (Fig. 3c, *p* ≤ 0.001). This result shows that intact infectious microvesicles accelerate RNA production in host cells at 3 hpi.

### Ultrastructural analysis by cryo-electron tomography revealed multiple classes of infectious microvesicles

**Figure 4:**
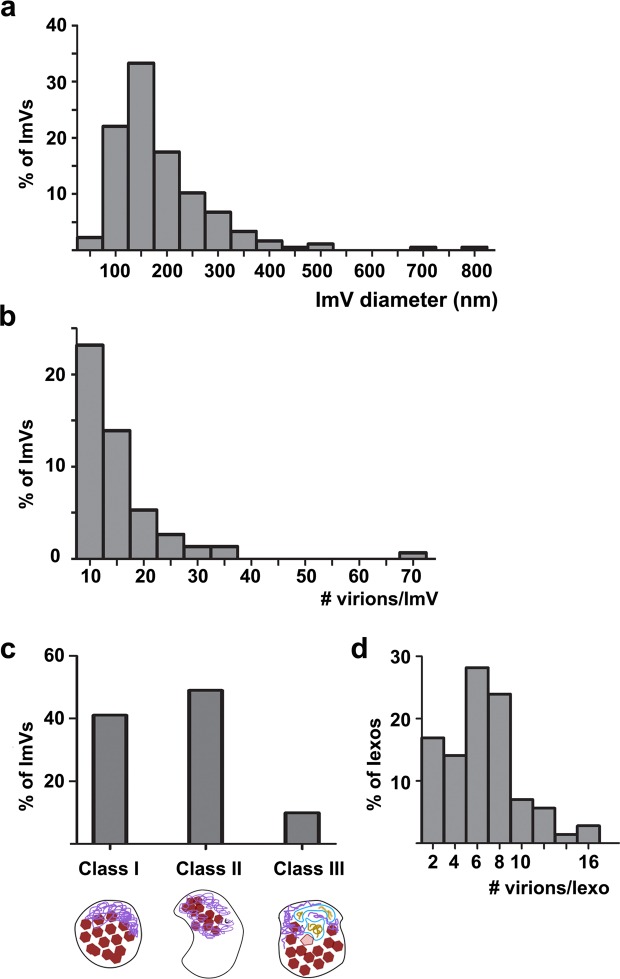
Statistics of vesicle size, virions per vesicles, and morphological classes of infectious extracellular vesicles from PV-infected cells. (**a**) Infectious microvesicle (ImV) diameter, (**b**) number of virions per ImV, (**c**) populations of morphological classes, analyzed from cryo-EM and cryo-ET data. As described in the text, Class I vesicles are close-packed with virions and additional electron-dense material termed “mat-like” structures because their density resembles a mat (or small rug); Class II vesicles are polar structures, with significant regions of low electron density in addition to virions, and mat-like densities; Class III vesicles contain inner vesicular structures in addition to virions and mat-like densities, and (**d**) number of virions per infectious exosome (Iexo).

A remaining unanswered question was the 3-D structures of virus-induced extracellular vesicles that, as shown above, accommodate not only viral capsids, but also unencapsidated viral RNA, cellular proteins, and viral replication proteins. To visualize vesicle ultrastructure, PS-positive infectious microvesicles and purified CD9-positive exosomes secreted by PV-infected cells from 4 to 8 hpi were preserved by plunge-freezing, and then imaged by cryo-EM and cryo-ET. To enrich the infectious extracellular vesicle population for low throughput cryo-EM, we imaged PS-positive microvesicles and CD9-positive exosomes. PS-positive infectious microvesicles showed a wide size distribution, with diameters ranging from 70 nm to 820 nm. Approximately 90% of these microvesicles were 100 to 300 nm in diameter, with a median of 170 nm (n = 210 vesicles, Fig. 4a). The microvesicles displayed neither a strong uniformity in size, nor a strong correlation between size and number of included virions. 3-D reconstructions computed using cryo-ET tilt series revealed that 70% of the vesicles (n = 180 vesicles) contained virions. Quantification of virions per infectious microvesicle shows that 83% of infectious microvesicles carry 1 to 20 virions and, on average, each infectious microvesicle transports 10 virions (n = 150 vesicles from cryo-ET data, Fig. 4b).

**Figure 5:**
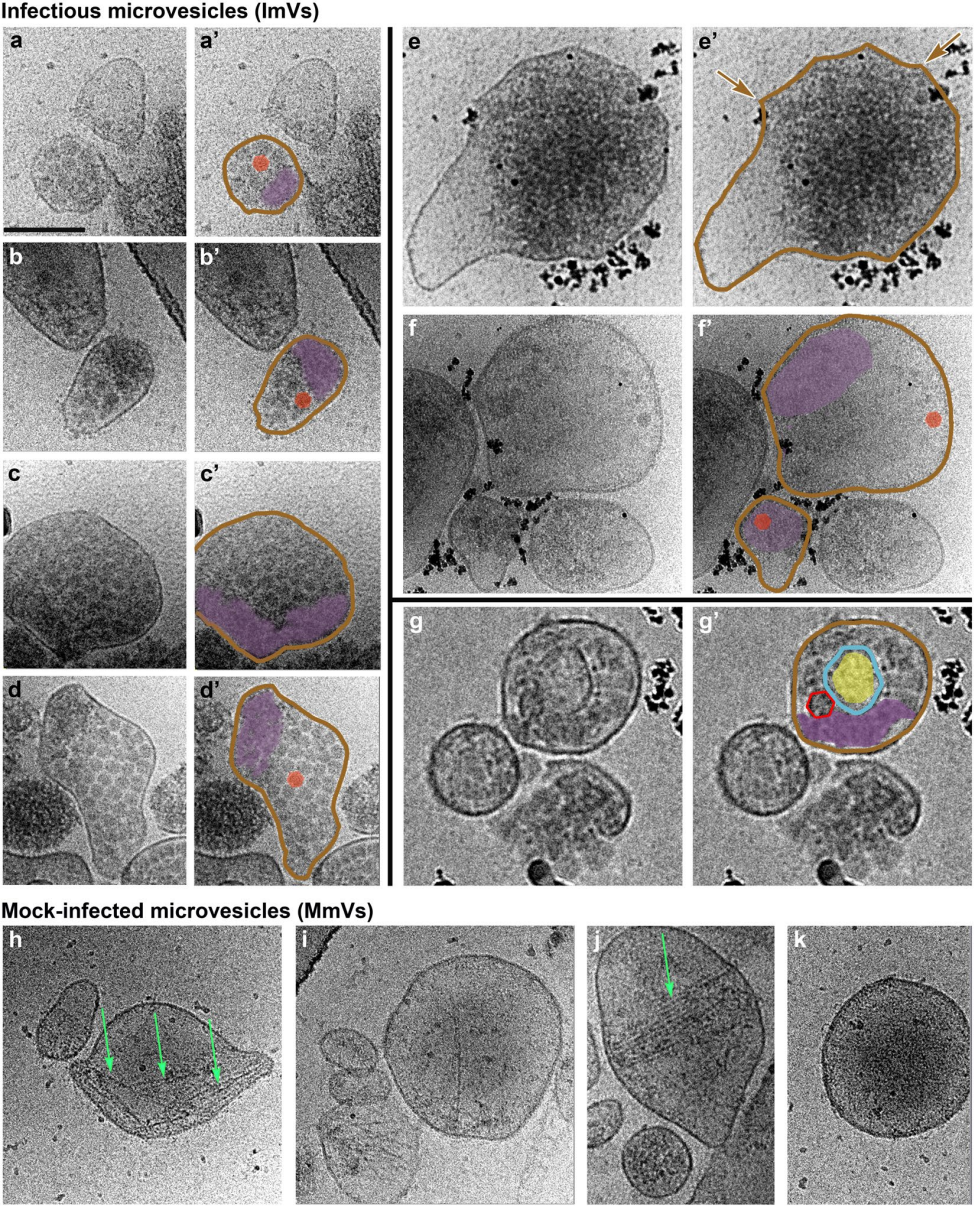
Virions and additional densities are visible in 2-D cryo-EM images of three morphological classes of infectious microvesicles secreted from PV-infected cells. (**a**–**d**) Class I vesicles are close-packed with virions and additional electron-dense material, termed “mat-like” structures because their density resembles a mat (or small rug). (**e–f**) Class II vesicles have similar internal densities, but are polar structures with significant regions of low electron density. (**g**) In addition to virions and mat-like structures, Class III vesicles contain inner vesicular structures. (**h–k**) Microvesicles from mock-infected cells have no virions, low internal density, and often include actin filaments (e.g., green arrows). Panels with primes highlight features including vesicle membranes (brown), virions (solid red), empty virions (traced in red, unfilled), mat-like density (purple), and inner vesicular structures (blue) with internal density (yellow). The outer membranes of infectious microvesicles often show a scalloped feature; e.g., region between brown arrows in **e’**. Scale bar, shown in **a,** represents 100 nm for all images. All images were taken at a tilt angle of 0 ° except the image in panel g, which was taken at a tilt angle of 15 ° to show the presence of an inner vesicle.

**Figure 6:**
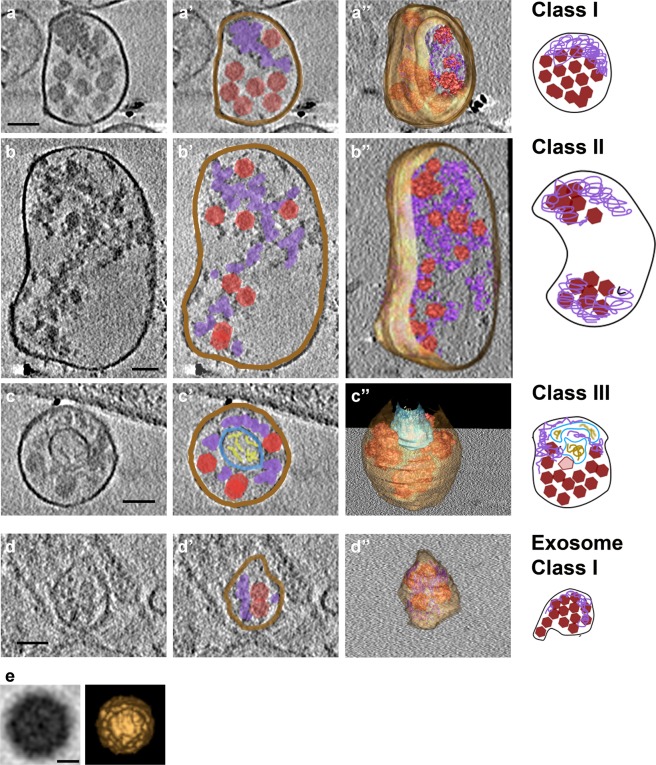
Three-dimensional data provide details and architectures of extracellular vesicles and virions from PV-infected cells. (**a–d**) Slices through tomograms computed from cryo-electron tomography (ET) tilt series of vesicles isolated at 8 hpi show details of the 3 classes of infectious microvesicles and infectious exosomes. (**a’–d’**) Features within the microvesicles and exosome are drawn on each slice, including virions (red polygons), mat-like density (purple), and inner vesicular structures (blue/yellow). (**a”–d”**) Isosurfaces of the membranes and mat-like densities are shown, and averaged virions (see panel e) are placed at each location where a virion is visible. The corresponding cartoon models are displayed on the right. (**e**) Sub-volume average of virions identified inside tomograms of infectious microvesicles and exosomes. Shown on the left, a low-pass filtered (spatial frequency cutoff = 0.028 Å^−1^) tomographic slice of the sub-volume average of 118 virions and on the right, an isosurface rendering of the sub-volume average. Scale bars (**a-d**) 100 nm; (**e**) 10 nm.

Based on their internal morphology, we categorized infectious microvesicles into three classes (Fig. 4c). As shown in cryo-EM images in Fig. 5, Class I contained densely packed virions (Fig. 5a–d), Class II contained clustered virions and low density regions (Fig. 5e–f), and Class III included internal vesicular structure(s) (Fig. 5g). In addition to virions, all classes contained high density material that resembles a mat of threads or noodles (modeled in purple in Figs. 5 and 6). As with size versus number of virions per infectious microvesicle discussed above, there was no correlation between infectious microvesicle size and the packing arrangement of virions (i.e. infectious microvesicle class). For example, a relatively large infectious microvesicle with a diameter of 200 nm could have Class I morphology (Fig. 5c,d) or Class II features (upper right vesicle in Fig. 5e,f).

As expected, no virions were observed within any vesicles isolated from mock-infected (control) cells (Fig. 5h–k). In contrast to infectious microvesicles, the major recognizable structural components observed in control microvesicles were disordered or bundled actin filaments (Fig. 5h–j, green arrows), which is consistent with the mass spectrometry data showing an abundance of actin and actin-binding proteins in microvesicles secreted from mock-infected cells (Table S1). In contrast, actin filaments were rarely observed within infectious microvesicles; in the few examples where infectious microvesicles contained actin filaments, multiple virions were dispersed within the bundled actin (Supplementary Fig. S5).

**Figure 7:**
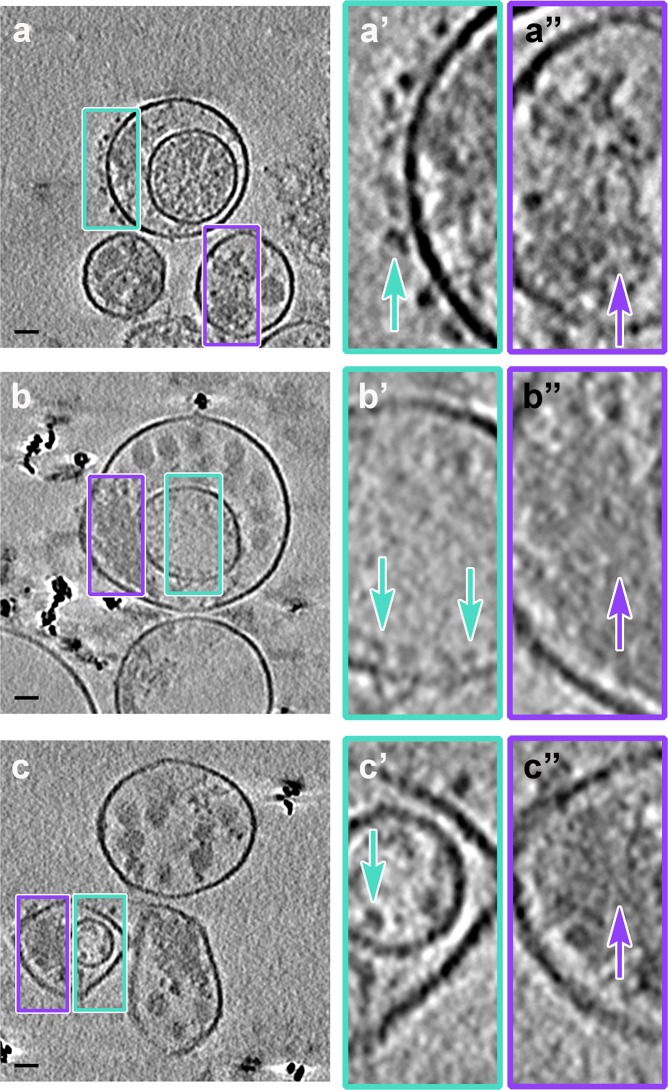
Mat-like densities are morphological features of extracellular vesicles from PV-infected cells. (**a–c**) Cryo-electron tomographic slices (8.2 nm thick) illustrate details of infectious microvesicles. Mat-like densities are present in all three classes of infectious microvesicles, purple arrows. Membrane-associated proteins are seen with internal or external globular domains, cyan arrows. EM Databank IDs: EMD-7877, EMD-7878, EMD-7873, respectively. (**a’–c’**) are the boxed regions of (**a–c**) at 3x magnification. Scale bars for original images, 100 nm; 33 nm for prime images (e.g. **a’**).

Three-dimensional reconstructions were computed using cryo-ET data from PS-positive infectious microvesicles and purified CD9-positive exosomes secreted by PV-infected cells from 4 to 8 hpi. Virions and density with a “mat-like” morphology were observed in the lumen of the microvesicles and exosomes (Figs. 6, 7, and Supplemental Movie). To confirm the presence of virions within the vesicles, we computed a subtomogram average of 118 structures from 1,022 particles we identified as virions, resulting in a reconstruction of the PV capsid at 6.9 nm resolution (Fig. 6e). Consistent with data from our 2-D cryo-EM images (Fig. 5), over 90% of the 3-D reconstructed infectious microvesicles showed either the Class I (Fig. 6a, deposited in the wwPDB under accession code EMD-7873) or Class II (Fig. 6b, wwPDB accession code EMD-7872) morphology, whereas Class III vesicles comprise only 10% of the population (Fig. 6c, wwPDB accession code EMD-7871, and Supplemental Movie). Infectious microvesicles were rarely spherical, and showed a single membrane enclosing an irregularly shaped structure, often with a scalloped outer contour, as seen in Fig. 5e,e’, (between the brown arrows). Class II infectious microvesicles (Fig. 6b), showed “empty” (low density) regions encompassing up to 90% of the infectious microvesicle volume, in addition to virions and mat-like structures. Class III infectious microvesicles contained inner vesicular structures as shown in Figs. 6c and 7, each representing a single-membrane vesicle entrapped in the lumen of an infectious microvesicle. The average ratio of diameter of the inner vesicle to that of its infectious microvesicle carrier was 0.5 ± 0.06 (n = 17). Less dense features were observed within inner vesicles (e.g. yellow region in Fig. 6c), as compared to the above-described mat-like structures of infectious microvesicles (Fig. 7, purple arrows). This suggests that structures inside the larger compartment of infectious microvesicles and those within inner vesicles may be distinct from each other. We also observed protein structures with globular ‘heads’ on a stalk in the membranes of both ImVs and their inner vesicles (Fig. 7, cyan arrows).

3-D structural analysis of CD9-positive exosomes secreted by PV-infected cells displayed a relatively uniform size, with an average diameter of 80 nm ± 27 nm and an average of 7 ± 3 virions per exosome (Fig. 4d; n = 69; data from nine reconstructed tomograms). The predominant morphology of exosomes from infected cells corresponded to features seen in Class I infectious microvesicles, with a densely packed interior volume (Fig. 6d, and deposited in the wwPDB, accession code EMD-7879).

## Discussion

It has long been observed that non-enveloped viruses, such as those in the enterovirus genus, can propagate infection prior to lysis of host cells^35,36^. However, the mechanism for exit (and entry) of these viruses without disruption of the cell’s plasma membrane is not well understood. Only recently has unconventional secretion of virion-containing extracellular vesicles been described^7–10,37^. Delivery of such a ‘payload’ containing multiple virions has the potential to propagate infection that might otherwise be hindered by frequent detrimental mutations in the viral genome that are present due to the inherently error-prone replication of RNA^34,38^. Such a diversity of virions can be achieved by either increasing the number of infecting free virions per cell (MOI) or by entry of a vesicle containing multiple virions into one cell.

**Figure 8:**
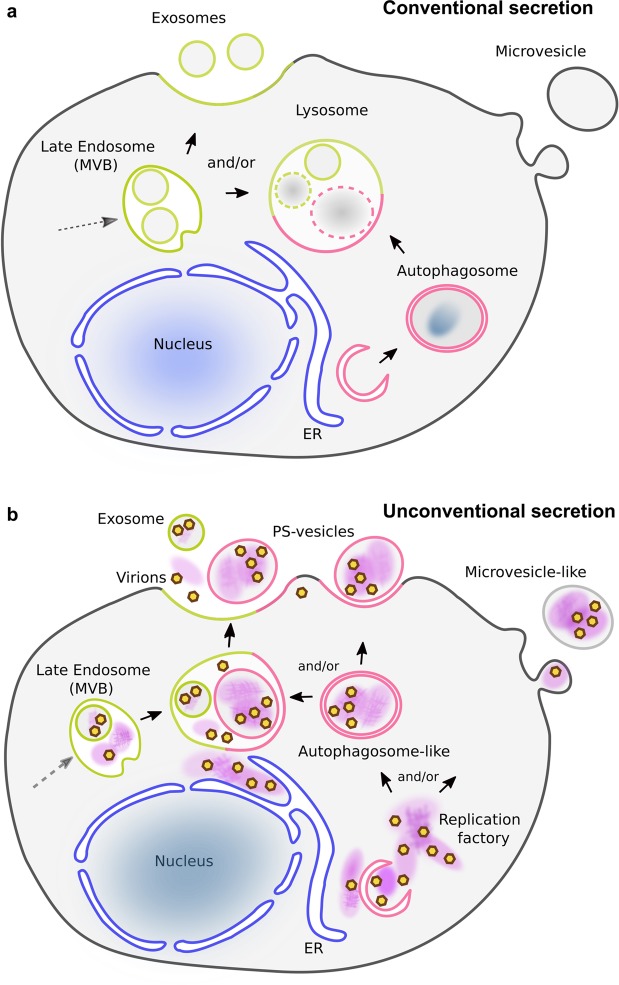
Models of cellular secretion from mock- and poliovirus- infected cells. (**a**) Endocytosis in uninfected cells provides a pathway for cell entry and distribution of internalized content to cell compartments. Cell sorting recycles selected components back to the plasma membrane while others become components of late endosomes, also called multivesicular bodies (MVBs). Components within the MVBs are then either exported as exosomes using (endosomal) ESCRT machinery, or fuse with lysosomes, where contents are degraded. Distinct from the endosomal pathway, autophagy is a mechanism to entrap and degrade specific cellular compartments and invading pathogens within double-membrane vesicles. Termed autophagosomes, these vesicles then fuse with lysosomes and the contents are degraded. In uninfected cells stimulated by, e.g. stress or starvation, lysosomes are the convergence point for the autophagic and endosomal pathways. (**b**) As PV infection progresses, membrane remodeling and lipid synthesis produce replication factories (purple patches) for vRNA synthesis. Prior to cell lysis, infectious exosomes and infectious microvesicles are formed within PV-infected cells. Infectious exosomes are present within late endosomes (also called multivesicular bodies, or MVBs; green). Exosomes are secreted either directly from MVBs, or after their fusion with autophagosome-like double membrane vesicles (pink). Infectious microvesicle-like structures can either bud directly from the cytoplasm, as in uninfected cells (see **a**), or they can be secreted after fusion of autophagosome-like vesicles with MVBs. Packaged inside exported vesicles are virions (brown/yellow hexagons), viral proteins, host proteins, host RNA, and both template and genomic viral RNA (together depicted as purple patches). These virion-containing extracellular vesicles get internalized into a neighboring cell. A rapid initiation of viral replication is achieved by this transport and internalization of all components needed for replication: virions, viral proteins, cellular proteins, ribosomes, viral RNA.

Here, we have presented data showing that in addition to infectious virions, extracellular microvesicles secreted from PV-infected cells contain a complex mixture of unencapsidated (+) vRNA as well as (−) vRNA, ready-made viral replication proteins and host proteins. Additionally, CD9-positive exosomes are involved in the non-lytic cell-to-cell transmission. In our new model, we speculate that vesicles comprising virions and macromolecules contribute to enhanced viral transmission and establishment of new infection, because in addition to virions, infected cells receive all the components necessary to begin infection *prior* to translation of the vRNA that is packaged into virions. A schematic of conventional cellular secretion (Fig. 8a) is compared to unconventional secretion, diagrammed by the interplay between viral replication and packaging of contents for non-lytic exit in Fig. 8b. In this figure we designate structures in infected cells as “autophagosome-like” and “microvesicle-like”, because while markers for these vesicles have been identified in PV-infected cells, it has not been unambiguously determined that these are either precisely autophagosomes or microvesicles.

### Vesicle components 1: viral RNAs and proteins

As has been shown previously^8,10,36^, we provide direct evidence that secreted infectious microvesicles transport virions from cell to cell (Figs. 3a, 5–6). We further show that exosomes are also infectious and involved in PV nonlytic cell-to-cell transmission (Fig. 3b). It has been proposed that multiple capsid-enclosed viral genomes that are transferred by vesicles *en bloc* into a single cell are the sole contributor to replication kinetics in the new host^10^. Indeed, this is the case for exosomes secreted from HAV-infected cells^37^. However, the delivery of (+) and (−) vRNA and viral replication proteins by extracellular vesicles into cells suggests that viral replication of PV may be facilitated by the delivery of (+) RNA, (−) RNA, and the viral proteins necessary for replication (e.g. the virus polymerase 3Dpol) together into new host cells. Support for these delivered components facilitating initial viral replication is our RT-qPCR data (Fig. 3c) showing an increase in (+) vRNA production in host cells at 3 hpi that is faster than infection with the same number of free naked virions^34^. However, because there are data showing a dependence of viral replication on *cis*-translation of a central region of the PV genome^39^, the question arises of roles for vesicle-transported non-structural proteins in *trans*. Perhaps the proximity of the proteins and RNA upon delivery overcomes the requirement of new translation, by eliminating the problem of diffusion-limited localization, such that the proteins are positioned as they would be, were they newly synthesized. This could result in production of a specific quaternary structure of vRNA necessary for replication^39^, providing a possible mechanism for subverting the *cis*-translation requirement for replication. Additionally, it remains to be explored whether this intact-ImV-induced jump-start to replication results in an overall increased vRNA production throughout the whole infection.

### Vesicle components, 2: host proteins

For infection propagation, viruses alter cell processes, facilitating the viral life cycle. This includes utilization of host cell proteins as well as ribosomes for translatation of viral proteins. Examples include 1) virus-induced reorganization of the actin cytoskeleton for viral entry^40,41^ and for transport of viral components within the cell^42^, and 2) utilization of host proteins during PV infection to shut down cap-dependent protein translation, so that protein production continues almost exclusively for (cap-independent) viral proteins^43^. With less than 10% of the proteins identified by mass spectrometry in secreted infectious vesicles being virus proteins, transported host cell proteins may have roles in advancing PV infection after cell-to-cell spread by vesicles. For example, our mass spectrometry data identified a significant enrichment of proteins from the glycolytic pathway in infectious microvesicles (p < 10^−8^, Table 1) that were not present in control microvesicles, including a strong and unique presence of pyruvate kinase PKM, the critical glycolysis rate-limiting enzyme. It is known that the presence of glucose during PV infection causes an average 170-fold increase in viral output^44^. Thus, a vesicle-infected host cell that has received PKM may be better prepared for viral replication than cells infected by free virions or infectious exosomes; PKM may assist in producing the large increase in glucose (or fructose) and glutamine known to be required for maximal PV replication^44,45^.

### Structures of infectious vesicles

Structure and function are often linked. Therefore, it is not surprising that after we identified the large number of components in infectious microvesicles and exosomes using biochemical methods, we were also able to visualize significant structural complexity in infectious vesicles that clearly contained internal components in addition to virions. Extracellular vesicles from PV-infected cells included dense structures with a mat-like morphology (Figs. 5–7), often seen adjacent to membranes, and/or in close proximity to virions. This juxtaposition suggests a possible involvement of protein-lipid interactions that results in the observed spatial arrangement of the cargo. While identification of the components that comprise the mat-like structures is not yet known, in our model these mats are composed of the non-capsid proteins identified by mass spectrometry (Table S1) and western blots (Fig. 1d); and vRNA identified by RT-qPCR (Fig. 2a). We note that unencapsidated (+) and (-) vRNA could be present as dsRNA, which would provide additional protection from the host immune system. Class III infectious vesicles, which are defined by the presence of internal membrane structures within the infectious microvesicle lumen, comprised only a minor population. It is currently unclear if Class III infectious vesicles are produced accidentally when a smaller vesicle happens to become entrapped, or if they originate from a cellular mechanism similar to the formation of multivesicular bodies, where the inclusion of additional components advances virus replication.

### Cellular origins of infectious vesicles

Cellular transport in uninfected cells, diagrammed in Fig. 8a, is altered in response to intracellular stress, such as an invading pathogen^46^. Autophagosomes can fuse with early or late endosomes/MVBs to form amphisomes^47^, and unconventional secretion can occur via secretory autophagy^48^ or lysosomal exocytosis^49^. Therefore, the endosomal, exosomal, lysosomal, and autophagic pathways are overlapping under some cellular conditions. Thus, it is not surprising that we identified both endocytic and exosomal marker proteins in infectious vesicles. We did not, however, identify autophagosome-associated lipidated LC3 (LC3-II). LC3-II was previously shown within cells, in autophagosome-like double membrane vesicles that contain virions; these autophagosomes are thought to be the precursor of secreted infectious vesicles^7–10,36^. This omission in our results, and previously in exosomes from HAV-infected cells^37^, is likely due to technical limitations in detecting lipidated proteins by LC-MS^50^.

In our model (Fig. 8), the endosomal and autophagosomal pathways both feed into and provide exits from viral replication factories that then utilize unconventional secretion for non-lytic spread of infectious vesicles, thereby accelerating viral replication in new cells.

### Summary

In summary, our results reveal that infectious vesicles derived from non-enveloped picornavirus-infected cells are a previously underappreciated viral transmission model. In this model, multi-component transport of virions, viral RNA, viral proteins and host proteins within microvesicles and exosomes facilitate cell-to-cell spread of viral infection by the non-enveloped virus PV.

## Materials & Methods

### Cell culture and PV infection

HeLa cells (ATCC, Manassas, VA) were infected with Mahoney PV (gift from Dr. Karla Kirkegaard, Stanford University) using protocols from Burrill, Strings, and Andino^51^. Briefly, HeLa cells were cultured in low-glucose DMEM medium supplemented with 5% fetal bovine serum (FBS) and 1% penicillin-streptomycin-glutamine (supplDMEM; Atlanta Biologicals, Cat. S11150; ThermoFisher, Cat. 10378016, respectively) and grown at 37 °C (5% CO_2_) to 60 to 80% confluence. After three washes with PBS + (PBS supplemented with 0.01 mg/ml MgCl_2_ and 0.01 mg/ml CaCl_2_), cells were infected either with PBS + or PBS + and PV stock at a multiplicity of infection (MOI) of 30 virions per cell, titrated by classic plaque assay (see below). After 30 min at 37 °C, cells were washed in PBS + and grown in supplDMEM. For the infectious microvesicle-induced infection assay shown in Fig. 3, infection was first synchronized by addition of infectious microvesicles on ice for 30 min to promote adherence, then washed with PBS+ , as in^52,53^ prior to growth at 37 °C in supplDMEM. Because PV replication and release of infectious extracellular vesicles decreased when cells were cultured in non-bovine serum media^51^, we first infected HeLa cells in FBS-containing media for 4 h, during which the RNA replication rate reaches its maximum. We then minimized contamination of the extracellular vesicles by components from bovine serum (FBS) by washing (3×) and then growing the cells in non-FBS media before collecting vesicles at 8 hpi, when cells and/or the supernatant were collected for subsequent analyses.

To test for cell viability at the time of vesicle collection (Fig. 1b), HeLa cells were infected with PV at an MOI of 30 for 8 h, then stained *in situ* with a 0.2% Trypan Blue solution in PBS. Persistently PV-infected K562 cells were collected and stained with a 1:1 dilution of the cell suspension in 0.4% Trypan Blue-PBS. In each case, samples were double-blinded and two randomly selected fields of approximately 200 cells from each of three replicate wells were counted. Percentage viability was calculated as number of blue-staining cells divided by the total cells counted x 100.

### Collection and purification of microvesicles and phosphatidylserine-containing microvesicles

The non-FBS media supernatant of control (mock-infected) or PV-infected HeLa cells at 8 hpi was collected as described above, and infectious microvesicles were purified as in^10^. Briefly, all at 4 °C, the supernatants were spun down at 150 × g for 15 min and then 2,000 × g for 20 min to remove cell debris and apoptosis bodies, respectively. The supernatant was then centrifuged at 10,000 × g, 60 min, to collect the extracellular vesicles with a size range of 100 nm to 1 μm in diameter, as in^17^. Both the supernatant and pellet were saved. For all experiments except LC/MS, the annexin-V microbead kit (Miltenyi Biotec, Cat. 130-090-201) was used for further enrichment of the pelleted PS-containing vesicles, per manufacturer’s instructions. The eluted PS-enriched vesicles were spun down and resuspended in 1X PBS for subsequent assays.

### Collection and purification of CD9-positive exosomes

To isolate CD9-positive exosomes, supernatants from the previously described 10,000 x g centrifugation were spun down at 100,000 x g for 1 h, the pellet was washed with 1X PBS, and spun again at 100,000 x g., three times. The final pellet was resuspended in 1X PBS, and exosomes were collected using ExoQuick-TC (System Biosciences Inc.) as per manufacturer’s instructions. The collected exosomes were further purified with anti-CD9 antibody-coated magnetic beads (CD9 Exo-Flow Exosome Purification Kit, System Biosciences, Cat. EXOTC10A-1) as in^54,55^.

### Sample preparation of broken extracellular vesicles

Extracellular vesicles were freeze-thawed (3×) to break vesicle membranes. For experiments in which RNA was to be removed, freeze-thawed vesicles were incubated with 1% sodium deoxycholate (300 μg/ml) on ice for 45 minutes as in^56^. The sample was then incubated with RNaseA (600 μg/ml, Sigma Cat. 10109142001) for 1 h at 37 °C, to remove exposed RNA, as in;^57^ at the 150 mM salt concentration in these experiments, we expect both ssRNA and dsRNA to be removed^58^.

### Immunoblotting

Purified extracellular vesicles were washed with 1X PBS, disrupted with chilled RIPA buffer (50 mM Tris/HCl pH 7.4, 75 mM NaCl, 1 mM EDTA, 1% NP-40, 0.25% sodium deoxycholate, 0.1% sodium dodecyl-sulfate) containing a protease inhibitor cocktail diluted 1:200 (Sigma, # P1860), and then incubated shaking for 30 min at 4 °C. Protein concentrations were determined by bicinchoninic assay (BCA, Thermo Fisher, # 23225). Five antibodies were used for immunoblotting: 1) anti-PV (type 1–3) polyclonal goat IgG (Abcam, # ab22450); 2) anti-3Dpol rabbit IgG prepared by Cocalico Biologicals from purified 3Dpol protein, 1 mg/ml, diluted 1:1000; 3) anti-2C (CPSQEHQEILFN) rabbit IgG (Cocalico Biologicals, 1 mg/ml, diluted 1:1000); 4) anti-3A (KDLKIDIKTSPPPEC;^59^) rabbit IgG (Biomatik, 0.6 mg/ml, diluted 1:500); 5) anti-GAPDH polyclonal rabbit IgG (Novus Biologicals # NB100–56875). Protein derived from extracellular vesicles (20 μg per lane) was subjected to SDS-PAGE or Tricine-SDS gel electrophoresis and western blotting, as described previously^60^.

### RT-qPCR

RNA from whole cells or extracellular vesicles was extracted using the RNeasy Plus Mini Kit (Qiagen, # 74134), as in^61^. The full-length PV genome and its negative-sense template were amplified, in duplicate, through two-step RT-qPCR, as previously described^51^. Briefly, cDNA was synthesized using the SuperScript III RT (Life Technology, # 18080-093) system; RT^34^, with Tag primer to increase binding specificity, full-length production, and efficiency. The qPCR was performed using a master mix (Fast SYBR Green master mix system; Life Technology, # 4385610). Detection of RNA was performed in a two-step RT-qPCR using the High Capacity cDNA Reverse Transcription Kit (Applied Biosystems, # 4374966) and the SYBR green master mix. In order to demonstrate the specificity of amplification, we conducted a series of controls including negative reverse transcription control and non-template controls, and melt curve analyses. As described in^62^, results were normalized to an endogenous control GAPDH of whole cells presented as ΔC_t_ (where ΔC_t_ = (C_t_ of endogenous control gene (GAPDH)) – (Ct of gene of interest))^62,63^. The mean of two technical replicates per cDNA sample was used to obtain raw C_t_ and ΔC_t_ for quantitative gene expression. The statistical analysis was obtained from six biological replicates. A non-paired one tailed Student’s t-test was used for the independent sample comparison, e.g. PV-infected (ImV) versus mock-infected (MmV) groups. A paired one tailed Student’s t-test was performed for comparison between the groups of untreated ImV/MmV and FTDR treated ImV/MmV. The Bonferroni method was used to calculate the *p*-value (cutoff = 0.05). The primer sequences for the following genes are listed below: PV (+) stranded_RT (GGCCGTCATGGCGAATAATGTGATGGATCCGGGGGTAGCG), PV (−) stranded_RT (GGCCGTCATGGTGGCGAATAACATGGCAGCCCCGG AACAGG), PV (+) stranded_F (CATGGCAGCCCCGGAACAGG), PV (−) stranded_R (TGTGATGGATCCGGGGGTAGCG), PV Tag (GGCCGTCATGGTGGCGAATAA), GAPDH_F (GCATCCTGGGCTACACTGAG), GAPDH_R (CCCTGTTGCTGTAGCCAAAT).

### Plaque assay

Virus titrations were performed as described previously^9,51^. Briefly, HeLa cells were seeded to a concentration of 2.0 × 10^6^/60 mm diameter tissue culture dishes and grown overnight. Serial dilutions of extracellular vesicles were then added, in duplicate, onto the cells for 30 min at 37 °C, 5% CO_2_, then overlayed with 1% agar in supplDMEM, and incubated 48 h. After fixation with 2% formaldehyde, 0.25% crystal violet stain was added for 10 min and rinsed in water. Plaques were counted manually.

### Plunge freezing for cryo-electron microscopy

Vesicle samples were freshly prepared for each experiment. C-Flat 4/2 holey carbon grids (Protochips, # CF-4/2-2C-50) were stabilized with an extra layer of carbon on the carbon surface. A 3 μl sample of extracellular vesicles was applied onto freshly glow-discharged grids. Samples for electron tomography included 0.5 μl of 5-nm fiducial gold (Ted Pella Inc. Cat. 82150-5) applied to the sample drop, and incubated for 1.5 minutes at 10 °C, 100% humidity. The grid was then blotted and plunge-frozen in a Vitrobot Mark III (FEI, Oregon), and stored in liquid nitrogen.

### Cryo-electron tomography

Initial cryo-electron microscopy was performed on a Philips CM12 EM at 100 kV (TVIPS CCD camera, pixel size of 6.8 Å, electron dose ≤ 30 e^−^/Å^2^). To collect data for 3-D reconstructions, the majority of the single axis tilt series (typical tilt range −56° to + 56°, angle increment 2°, total electron dose ≤ 100 e^−^/Å^2^, defocus of −4 to −5 μm, were acquired on an FEI Tecnai F20 (TF20) EM at 160 kV (TVIPS CMOS camera; 2x binned pixel size of 8.3 Å) using SerialEM software^64^. Alternatively, some single axis tilt series were collected on a JEM JEOL 2200 FS equipped with an in-column Omega energy filter, at 200 kV on a Direct Electron Detector (DE20) using SerialEM software^64^, resulting in a pixel size of 4.01 Å. The typical tilts were recorded from −60° to + 60°, with an angle increment of 2°, total electron dose ≤ 100 e^−^/Å^2^, defocus of −4 μm. The tilt images collected on the DE20 were motion corrected using scripts provided by the manufacturer (Direct Electron, LP). Reconstructions of tomograms and 3-D surface model rendering were performed using the weighted back-projection method from the IMOD software package^65^. Volume averaging of virions inside vesicles was performed using PEET software^66,67^ and the 3-D surface model of virions was rendered through averaged and low pass filtered sub-volumes from smoothed tomograms. Nonlinear anisotropic diffusion (NAD) filtering was used to generate 3-D surface models of inner mat-like structures in the infectious microvesicles, to provide better density continuity and reduced noise.

### Subtomogram averaging

Subvolumes of virus-like particles inside ImVs were manually selected (n = 1,022) from 32 smoothed tomograms (data recorded on an FEI TF20 EM) using the IMOD software package to determine the initial orientation of each particle. Alignment and averaging were performed using the PEET software (which is part of the IMOD package). Classification analyses using principal component analysis (PCA) were performed on a set of aligned sub-volumes^67^ to group structurally homogeneous particles using clusterPca in PEET. The majority of virions (820 out of 1,022) were identified in class 1, with 160, 32, and 10 virions in each of the next three classes. Class 1 virions with high cross-correlation coefficients (118 particles) were averaged, including imposing icosohedral symmetry; the process was then iterated using the average as the new reference. Based on the resolution estimation using the Fourier Shell Correlation (FSC at 0.5 cutoff), the final subtomogram average was low-pass filtered to a resolution of 6.9 nm and each virion in the tomogram is displayed using isosurface-rendering of the symmetrized averaged volume.

### Liquid chromatography-mass spectrometry

Infectious microvesicles (without annexin-V purification) from mock- and PV- infected cells at 8 hpi were collected as described above. Collected vesicles were sonicated in an ice-water bath for 4 min, then incubated with 2,2,2,-Trifluoroethanol (TFE) (Sigma, # T63002) (1:1, V:V), for 2 h at 60 °C with shaking. Ammonium bicarbonate (J.T. Baker) and dithiothreitol (DTT) (Sigma) were added at room temperature (RT), to final concentrations of 50 mM and 5 mM, respectively, and incubated at 60 °C for 30 min. Iodoacetamide (Bio-Rad) was added to a final concentration of 10 mM, incubated 30 min at RT in the dark and then quenched with DTT. After dilution to 5% TFE with a 3:1 mixture of water:50 mM ammonium bicarbonate, sequencing-grade trypsin (Promega Corp. Madison, WI) was added at 1:30 enzyme to substrate (V:V), incubated overnight at 37 °C, quenched with neat formic acid and cleaned using C18 PepClean spin columns (Pierce Biotechnology, Rockford, IL).

Peptides from three technical replicates were analyzed by liquid chromatography–tandem mass spectrometry (LC-MS/MS) using a Q Exactive Hybrid Quadrupole-Orbitrap mass spectrometer (Thermo-Fisher) in positive mode, equipped with a nanoAcquity UPLC system, (nanoAcquity NPLC Symmetry C18 trap column and ACQUITY UPLC Peptide BEH C18 analytical column; Waters) and a Triversa Nanomate source (Advion, Ithaca, NY). Using mobile phase A (1% acetonitrile/0.1% formic acid) and mobile phase B (99% acetonitrile/0.1% formic acid), peptides were trapped for 4 min at 4 μL/min in A, and then separated using the gradient: 0–8 min: 2% B, 8–96 min: 2–40% B, 96–102 min: 40% B, 102–105 min: 40–70% B, 105–113 min: 70% B, 113–114 min: 70–2% B, and 114–120 min: 2% B. MS spectra were acquired at 70,000 resolution at *m/z* 400, scan range *m/z* 370–1800, 1 microscan per spectrum, AGC target of 1e6, maximum injection time 100 ms. The 10 most abundant precursor ions per scan were fragmented at 17,500 resolution at *m/z* 400, AGC target 1e6, maximum injection time 100 ms, isolation window 10.0 *m/z*, isolation offset 0.4 *m/z*, normalized collision energy (NCE) 26, exclusion of unassigned charge states and charge states 1, and dynamic exclusion 8 s. Profile data were recorded for both MS and MS/MS scans.

### Proteomics Data analysis

LC-MS/MS data were processed using Peaks Studio 8.5 (Bioinformatics Solutions, Waterloo, ON)^68^, using a database containing the PV proteome, either as one combined polypeptide or as individual polio proteins and stable intermediates, concatenated with human and bovine Uniprot proteomes^69^. Technical replicates were searched together. We specified precursor ion (MS1) error tolerance of 10 ppm, and a fragment ion (MS/MS) error tolerance of 0.02 Da and a target-decoy false discovery threshold of 0.1%. Proteins identified as exclusively bovine were discarded, and we required protein identifications to contain at least two unique peptides when searching against the PV proteome as one polypeptide, and at least one unique peptide for searches against individual polio proteins and stable intermediates. Protein abundances were calculated by aggregating the MS1 peak areas for all the peptides identified for a specific protein, and normalized to an endogenous control GAPDH of whole cells.

To identify cellular pathways that were significantly associated with the identified protein matches, p-values of overlap were determined by IPA software (Qiagen) using Fisher’s exact test. Pathways shown to be enriched (protein matches were over-represented in our datasets as compared to a random dataset of the cellular proteome) were included when –log(p-value) > 4.

### Schematic diagrams

Schematic diagrams were drawn using Inkscape open source software (inkscape.org).

## Supplementary information

Supplementary information.

Supplementary movie; Supplementary information 2.

## Data Availability

Cryo-ET data have been deposited in the wwPDB, with EMDB accession codes: EMD-7877, EMD-7878, EMD-7879, EMD-7880, EMD-7881.
