# Supplementary material for: Complexity and ultrastructure of infectious extracellular vesicles from cells infected by non-enveloped virus
Source: Sci Rep. 2020 May 14;10:7939. doi: 10.1038/s41598-020-64531-1 (PMC7224179; doi:10.1038/s41598-020-64531-1)
Supplement: Supplementary file 1 — Supplementary information. [file 41598_2020_64531_MOESM1_ESM.pdf]

Complexity and ultrastructure of infectious extracellular vesicles from cells infected by non-enveloped virus

Jie E. Yang[1¶], Evan D. Rossignol[1§], Deborah Chang[2], Joseph Zaia[2], Isaac Forrester[3], Kiran Raja[1❖], Holly Winbigler[4], Daniela Nicastro[5], William T. Jackson[4], Esther Bullitt[1#]

Supplementary Figure 1. Viral Proteins and vRNA expression level in whole cells.

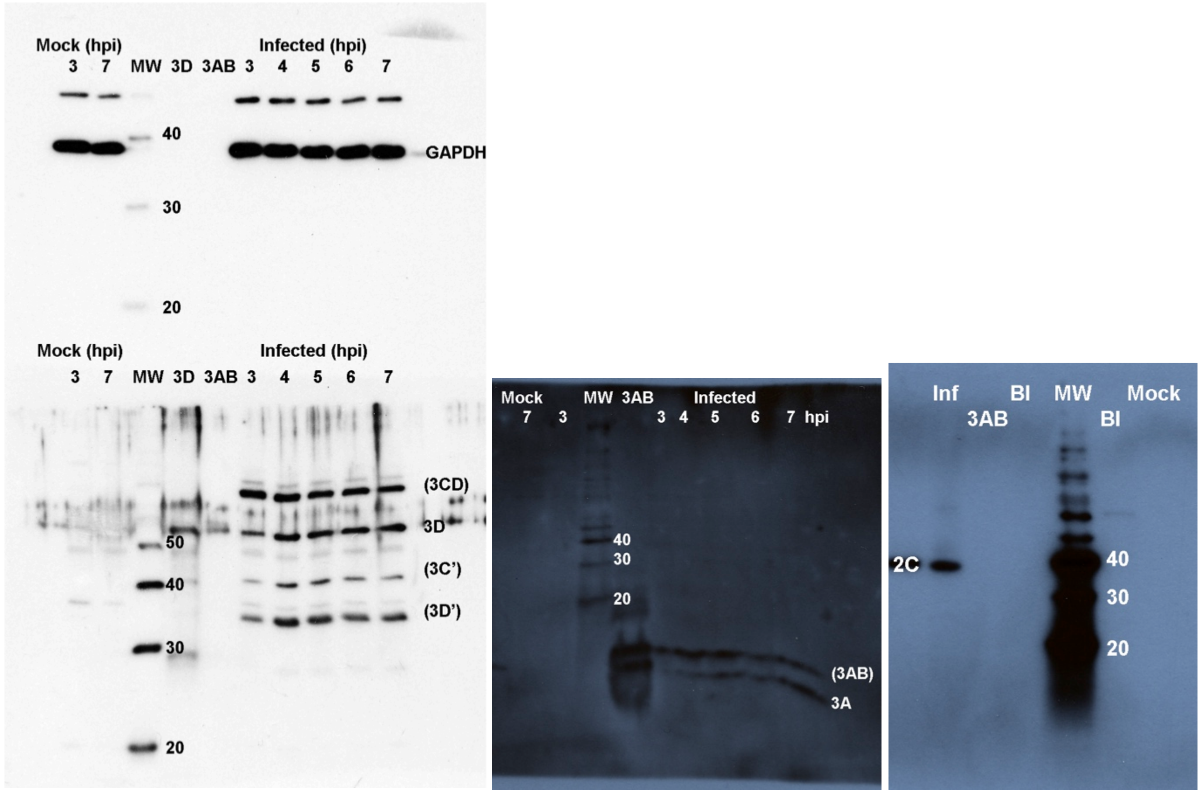

(a) (b) (c)

Supplementary Figure 1. Viral Proteins and vRNA expression level in whole cells.

(a) Proteins from lysates of PV- and mock- infected cells at 3, 4, 5, 6, and 7 hours post infection (hpi) were probed by western blot. The blot was first probed for GAPDH (top panel) to show similar loading in all lanes. The blot was then stripped, and re-probed using polyclonal anti-3D antibodies. All species containing the 3D protein are expected to be labeled by the anti-3D antibody (e.g. Garmarnik & Andino (1998). Genes and Development 12:2293-2304, and Teterina et al., (2001). J. Virology 75:3841-3850); these additional species are shown in parentheses.

(b) Proteins from lysates of mock-infected (3 and 7 hpi) and PV-infected cells (3, 4, 5, 6, and 7 hpi) were probed for PV 3A; it is expected that 3AB is also labeled by this antibody, as indicated by parentheses.

(c) Proteins from lysates of PV-infected (Inf) and mock-infected (Mock) cells at 7 hpi were probed for PV 2C.

3AB: purified PV 3AB protein
Bl: blank lanes
MW: molecular weight markers.

Supplementary Figure 2
Full Western Blots, probed with polyclonal antibodies against (a) VP0-VP3; (b) 2C; (c) 3D; (d) 3A

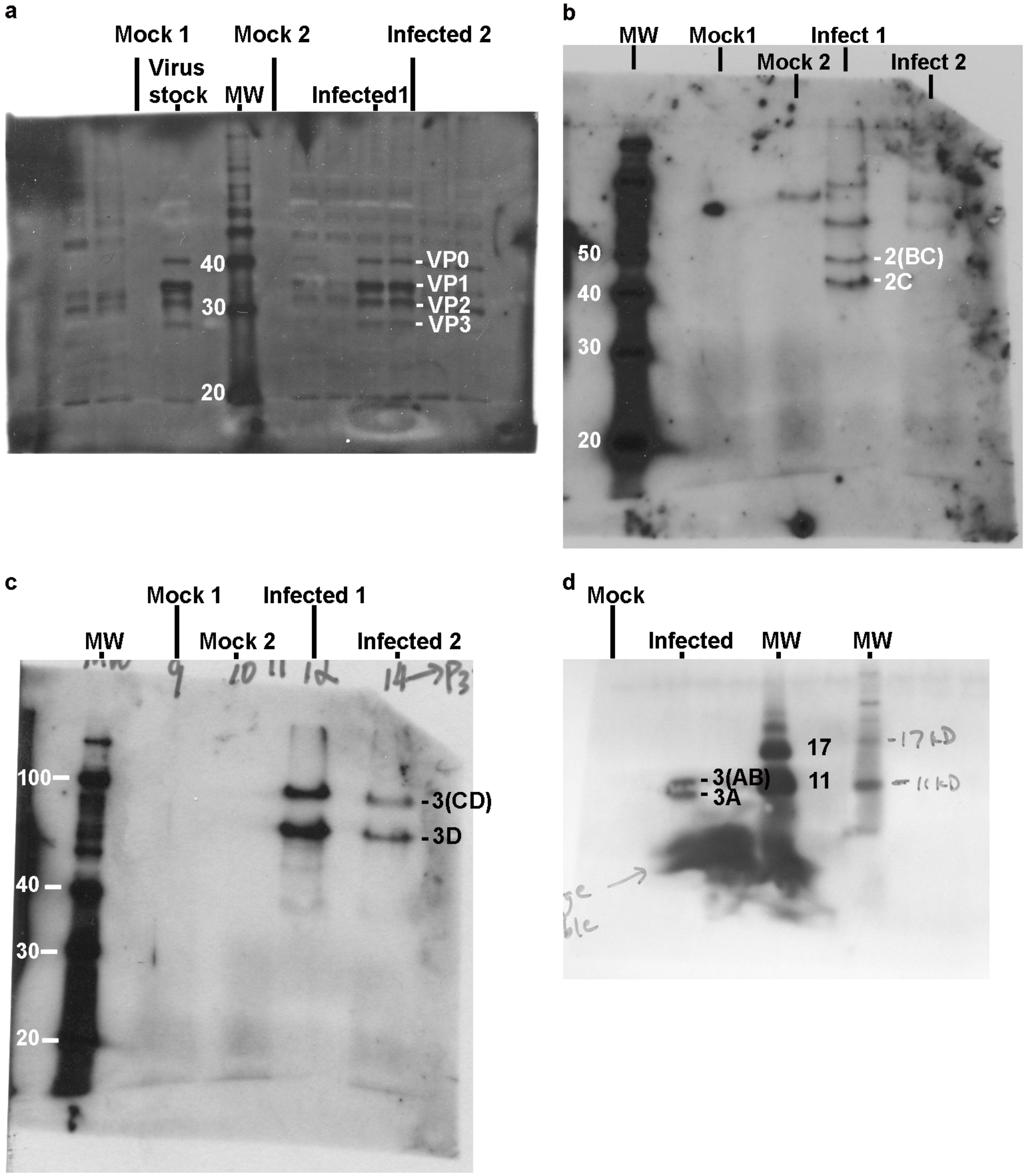

Supplementary Figure 2. Viral proteins are present in vesicles secreted by virus-infected cells.

Full western blots from Figure 1d, showing the presence of viral proteins in microvesicles secreted by PV-infected HeLa cells at 8 hours post infection (hpi), but not in vesicles isolated from mock-infected cells.

Vesicles were run on tricine gels, and probed with (a) anti-VP capsid protein antibodies; the membrane on the left is shown for its molecular weight markers (MW) and poliovirus stock

solution, (b) anti-C antibodies; Mock 1 (vesicles from mock-infected cells) and Infect 1 (vesicles from PV-infected cells) are from one experiment, and Mock 2 and Infect 2 are from an independent experiment, (c) anti-3D antibodies; Mock prep 1 (vesicles from mock-infected cells) and Infected prep 1 (vesicles from PV-infected cells) are from one experiment, and Mock prep 2 and Infected prep 2 are from an independent experiment, (d) anti-3A antibodies; lanes are samples of vesicles secreted by mock- and PV- infected cells at 7 hpi. All species containing the protein probed for are expected to be labeled by that antibody (see, e.g., Garmarnik & Andino (1998). Genes and Development 12:2293-2304, and Teterina et al., (2001). J. Virology 75:3841-3850); these additional species are shown in parentheses. MW: molecular weight markers.

Supplementary Figure 3 b-k (panel a is blank)

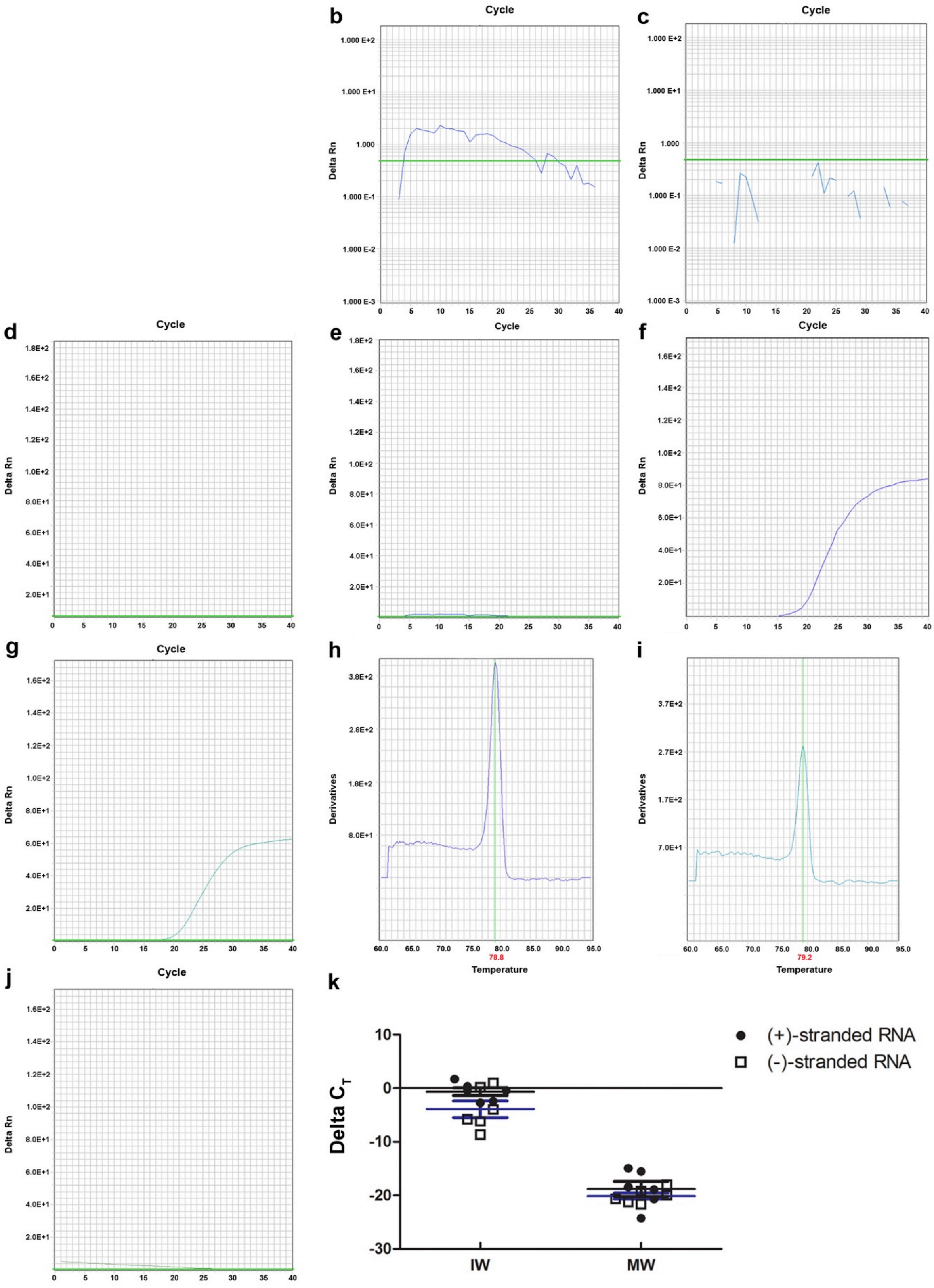

Supplementary Figure 3. **Viral Proteins and vRNA expression level in whole cells.**

(b-i) Confirmation and determination of specificity of the positive- and negative-strand RNA assays show no amplification in PV-infected cells when reverse transcriptase was absent, probed

using positive- (b) and negative- (c) sense RNA strands. No amplification was observed in water, probed using positive- (d) and negative- (e) sense RNA. Primers were able to specifically recognize positive- (f) and negative- (g) sense viral RNA from the total RNA extracted from PV-infected cells. The single peak in the melt curve of cDNA products of positive- (h) and negative- (i) sense viral RNA probes from PV-infected cells indicates the presence of one target amplicon. The amplification of positive-sense viral RNA was absent in mock-infected cells (j). The total RNA of PV- (IW) and mock-infected (MW) cells at 8 hpi was collected, and the levels of (+) vRNA and (-) vRNA were determined using RT-qPCR (k). The y-axis indicates fluorescent intensity signal in arbitrary units. Panels b, c are expressed in log mode. Panels d-j are expressed in linear mode. RT-qPCR relative quantification was calculated as ΔC_t where ΔC_t = (C_t of endogenous control gene (GAPDH)) – (Ct of gene of interest (vRNA)), using GAPDH of whole cells for normalization.

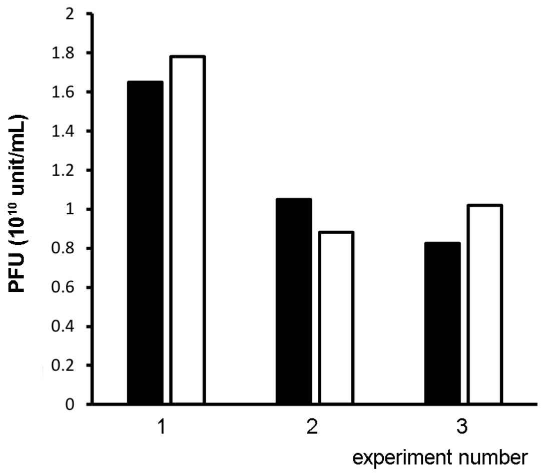

Supplementary Figure 4. PV infectivity before and after the treatment of freeze-thaw, detergent, RNAse.

The titer of poliovirus stock solutions was determined by plaque assay before (black) and after (white) freeze-thaw, detergent, and RNAse treatment. The y-axis indicates the viral titer (plaque-forming units) while the x-axis indicates three independent experiments.

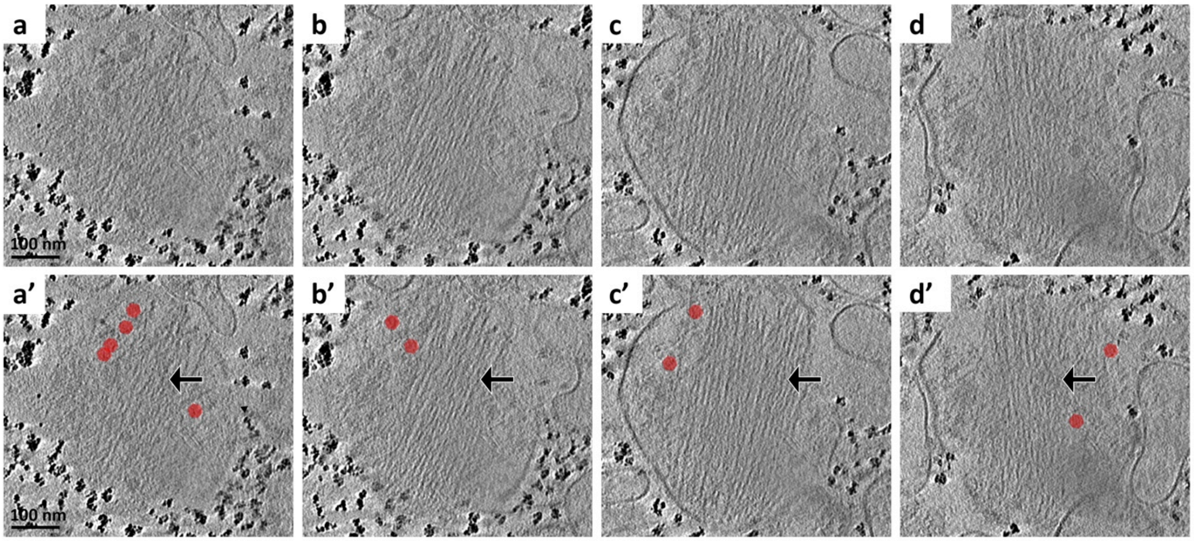

Supplementary Figure 5. Virions were rarely seen dispersed within the bundled actin filaments carried by infectious microvesicles.

Successive 1 nanometer thick slices through a reconstructed cryo-electron tomogram (a-d). Traced models of (a'-d') depict the disappearance and appearance of individual virions (red hexagons) among actin filaments (black arrows). The scale bar is 100 nm.

Supplemental Movie. Cryotomogram of a microvesicle from poliovirus-infected cells

Movie of an electron cryotomogram of a Class III microvesicle isolated from poliovirus-infected cells. Labeled, and later shown in isosurface-rendered models in the tomogram, are virions, vesicle membranes, mat-like structures, and the inner vesicular structure. Additional labels indicate representative annexin-V beads that were used to isolate the sample, and fiducial gold beads that were added to aid alignment of tilt series data. Scale bar, 100 nm.

Supplemental Table S1. Proteins Identified in Microvesicles Isolated from Cells that were:

PV-infected **Mock-infected**

Accession #	Protein Abbreviation	-10lgP	P	Aggregated Area from one run of ImVs	Description		Aggregated Area from one run of MmVs	-10lgP	P
P60709	ACTB_HUMAN	378.78	1.32E-38	975000000	Actin cytoplasmic 1 OS=Homo sapiens GN=ACTB PE=1 SV=1		25400000	139.43	1.14E-14
P63261	ACTG_HUMAN	378.78	1.32E-38	975000000	Actin cytoplasmic 2 OS=Homo sapiens GN=ACTG1 PE=1 SV=1		25400000	139.43	1.14E-14
P80723	BASP1_HUMAN	370.94	8.05E-38	288000000	Brain acid soluble protein 1 OS=Homo sapiens GN=BASP1 PE=1 SV=2				
P35908	K22E_HUMAN	330.12	9.73E-34	517000000	Keratin type II cytoskeletal 2 epidermal OS=Homo sapiens GN=KRT2 PE=1 SV=2		4230000	115.88	2.58E-12
P04264	K2C1_HUMAN	307.63	1.73E-31	488000000	Keratin type II cytoskeletal 1 OS=Homo sapiens GN=KRT1 PE=1 SV=6		19900000	150.49	8.93E-16
P50995	ANX11_HUMAN	269.92	1.02E-27	148000000	Annexin A11 OS=Homo sapiens GN=ANXA11 PE=1 SV=1				
P13645	K1C10_HUMAN	266.4	2.29E-27	416000000	Keratin type I cytoskeletal 10 OS=Homo sapiens GN=KRT10 PE=1 SV=6		14000000	141.52	7.05E-15
P35613	BASI_HUMAN	253.39	4.58E-26	24400000	Basigin OS=Homo sapiens GN=BSG PE=1 SV=2				
P02533	K1C14_HUMAN	237.36	1.84E-24	30700000	Keratin type I cytoskeletal 14 OS=Homo sapiens GN=KRT14 PE=1 SV=4				
P04406	G3P_HUMAN	227.96	1.60E-23	92700000	Glyceraldehyde-3-phosphate dehydrogenase OS=Homo sapiens GN=GAPDH PE=1 SV=3				
P11142	HSP7C_HUMAN	225.22	3.01E-23	14600000	Heat shock cognate 71 kDa protein OS=Homo sapiens GN=HSPA8 PE=1 SV=1				
P04083	ANXA1_HUMAN	218.34	1.47E-22	31700000	Annexin A1 OS=Homo sapiens GN=ANXA1 PE=1 SV=2				
P08195	4F2_HUMAN	214.05	3.94E-22	111000000	4F2 cell-surface antigen heavy chain OS=Homo sapiens GN=SLC3A2 PE=1 SV=3				
O75340	PDCD6_HUMAN	213.31	4.67E-22	213000000	Programmed cell death protein 6 OS=Homo sapiens GN=PDCD6 PE=1 SV=1				
P35527	K1C9_HUMAN	206.24	2.38E-21	178000000	Keratin type I cytoskeletal 9 OS=Homo sapiens GN=KRT9 PE=1 SV=3				
P23528	COF1_HUMAN	204.53	3.52E-21	19300000	Cofilin-1 OS=Homo sapiens GN=CFL1 PE=1 SV=3				
P05023	AT1A1_HUMAN	204.08	3.91E-21	25300000	Sodium/potassium-transporting ATPase subunit alpha-1 OS=Homo sapiens GN=ATP1A1 PE=1 SV=1				
P08174	DAF_HUMAN	190.48	8.95E-20	216000000	Complement decay-accelerating factor OS=Homo sapiens GN=CD55 PE=1 SV=4				
P13987	CD59_HUMAN	190.45	9.02E-20	61500000	CD59 glycoprotein OS=Homo sapiens GN=CD59 PE=1 SV=1				
P09525	ANXA4_HUMAN	181.51	7.06E-19	19800000	Annexin A4 OS=Homo sapiens GN=ANXA4 PE=1 SV=4				
P26038	MOES_HUMAN	178.96	1.27E-18	23500000	Moesin OS=Homo sapiens GN=MSN PE=1 SV=3				
P62937	PPIA_HUMAN	173.59	4.38E-18	40700000	Peptidyl-prolyl cis-trans isomerase A OS=Homo sapiens GN=PPIA PE=1 SV=2				
P60174	TPIS_HUMAN	164.69	3.40E-17	27000000	Triosephosphate isomerase OS=Homo sapiens GN=TPI1 PE=1 SV=3				
P30626	SORCN_HUMAN	157.35	1.84E-16	42900000	Sorcin OS=Homo sapiens GN=SRI PE=1 SV=1				
P07195	LDHB_HUMAN	156.21	2.39E-16	991000	L-lactate dehydrogenase B chain OS=Homo sapiens GN=LDHB PE=1 SV=2				
P61586	RHOA_HUMAN	152.91	5.12E-16	6180000	Transforming protein RhoA OS=Homo sapiens GN=RHOA PE=1 SV=1				
Q09666	AHNK_HUMAN	150.48	8.95E-16	7160000	Neuroblast differentiation-associated protein AHNAK OS=Homo sapiens GN=AHNAK PE=1 SV=2				
P08238	HS90B_HUMAN	150.15	9.66E-16	15800000	Heat shock protein HSP 90-beta OS=Homo sapiens GN=HSP90AB1 PE=1 SV=4				
P63104	1433Z_HUMAN	147.65	1.72E-15	12000000	14-3-3 protein zeta/delta OS=Homo sapiens GN=YWHAZ PE=1 SV=1				
P62987	RL40_HUMAN	144.04	3.94E-15	54000000	Ubiquitin-60S ribosomal protein L40 OS=Homo sapiens GN=UBA52 PE=1 SV=2				
P62979	RS27A_HUMAN	144.04	3.94E-15	54000000	Ubiquitin-40S ribosomal protein S27a OS=Homo sapiens GN=RPS27A PE=1 SV=2				
P0CG48	UBC_HUMAN	144.04	3.94E-15	54000000	Polyubiquitin-C OS=Homo sapiens GN=UBC PE=1 SV=3				
P06733	ENOA_HUMAN	142.93	5.09E-15	23900000	Alpha-enolase OS=Homo sapiens GN=ENO1 PE=1 SV=2				
P08758	ANXA5_HUMAN	142.1	6.17E-15	24600000	Annexin A5 OS=Homo sapiens GN=ANXA5 PE=1 SV=2				
P16070	CD44_HUMAN	141.89	6.47E-15	5710000	CD44 antigen OS=Homo sapiens GN=CD44 PE=1 SV=3				
O95274	LYPD3_HUMAN	141.14	7.69E-15	4970000	Ly6/PLAUR domain-containing protein 3 OS=Homo sapiens GN=LYPD3 PE=1 SV=2				
P02786	TFR1_HUMAN	140.98	7.98E-15	7890000	Transferrin receptor protein 1 OS=Homo sapiens GN=TFRC PE=1 SV=2				
P31949	S10AB_HUMAN	140.88	8.17E-15	52900000	Protein S100-A11 OS=Homo sapiens GN=S100A11 PE=1 SV=2				
P04075	ALDOA_HUMAN	140.39	9.14E-15	10700000	Fructose-bisphosphate aldolase A OS=Homo sapiens GN=ALDOA PE=1 SV=2				
P14618	KPYM_HUMAN	138.78	1.32E-14	17500000	Pyruvate kinase PKM OS=Homo sapiens GN=PKM PE=1 SV=4				

Supplemental Table S1 (continued).

Proteins Identified in Microvesicles Isolated from Cells that were:

PV-infected (continued)

Q8WUM4	PDC6I_HUMAN	138.08	1.56E-14	6960000	Programmed cell death 6-interacting protein OS=Homo sapiens GN=PDCD6IP PE=1 SV=1	
P20073	ANXA7_HUMAN	135.42	2.87E-14	13500000	Annexin A7 OS=Homo sapiens GN=ANXA7 PE=1 SV=3	
P32004	L1CAM_HUMAN	134.29	3.72E-14	5020000	Neural cell adhesion molecule L1 OS=Homo sapiens GN=L1CAM PE=1 SV=2	
P07437	TBB5_HUMAN	129.88	1.03E-13	4670000	Tubulin beta chain OS=Homo sapiens GN=TUBB PE=1 SV=2	
P15311	EZRI_HUMAN	129.76	1.06E-13	4090000	Ezrin OS=Homo sapiens GN=EZR PE=1 SV=4	
O75131	CPNE3_HUMAN	123.29	4.69E-13	10200000	Copine-3 OS=Homo sapiens GN=CPNE3 PE=1 SV=1	
P00338	LDHA_HUMAN	121.52	7.05E-13	7580000	L-lactate dehydrogenase A chain OS=Homo sapiens GN=LDHA PE=1 SV=2	
P05556	ITB1_HUMAN	120.67	8.57E-13	9540000	Integrin beta-1 OS=Homo sapiens GN=ITGB1 PE=1 SV=2	
P0DMV8	HS71A_HUMAN	118.29	1.48E-12	3530000	Heat shock 70 kDa protein 1A OS=Homo sapiens GN=HSPA1A PE=1 SV=1	
P0DMV9	HS71B_HUMAN	118.29	1.48E-12	3530000	Heat shock 70 kDa protein 1B OS=Homo sapiens GN=HSPA1B PE=1 SV=1	
Q9UBV8	PEF1_HUMAN	118.08	1.56E-12	25400000	Peflin OS=Homo sapiens GN=PEF1 PE=1 SV=1	
P10809	CH60_HUMAN	114.89	3.24E-12	3020000	60 kDa heat shock protein mitochondrial OS=Homo sapiens GN=HSPD1 PE=1 SV=2	
Q16658	FSCN1_HUMAN	112.1	6.17E-12	6460000	Fascin OS=Homo sapiens GN=FSCN1 PE=1 SV=3	
P08754	GNAI3_HUMAN	111.38	7.28E-12	4060000	Guanine nucleotide-binding protein G(k) subunit alpha OS=Homo sapiens GN=GNAI3 PE=1 SV=3	
O00299	CLIC1_HUMAN	96.21	2.39E-10	2990000	Chloride intracellular channel protein 1 OS=Homo sapiens GN=CLIC1 PE=1 SV=4	
Q9H444	CHM4B_HUMAN	95.61	2.75E-10	5080000	Charged multivesicular body protein 4b OS=Homo sapiens GN=CHMP4B PE=1 SV=1	
P08133	ANXA6_HUMAN	91.15	7.67E-10	1380000	Annexin A6 OS=Homo sapiens GN=ANXA6 PE=1 SV=3	
Q06830	PRDX1_HUMAN	90.78	8.36E-10	11500000	Peroxiredoxin-1 OS=Homo sapiens GN=PRDX1 PE=1 SV=1	
O00159	MYO1C_HUMAN	85.9	2.57E-09	1650000	Unconventional myosin-Ic OS=Homo sapiens GN=MYO1C PE=1 SV=4	
P68104	EF1A1_HUMAN	83.73	4.24E-09	19500000	Elongation factor 1-alpha 1 OS=Homo sapiens GN=EEF1A1 PE=1 SV=1	
Q05639	EF1A2_HUMAN	83.73	4.24E-09	19500000	Elongation factor 1-alpha 2 OS=Homo sapiens GN=EEF1A2 PE=1 SV=1	
Q5VTE0	EF1A3_HUMAN	83.73	4.24E-09	19500000	Putative elongation factor 1-alpha-like 3 OS=Homo sapiens GN=EEF1A1P5 PE=5 SV=1	
P15328	FOLR1_HUMAN	80.6	8.71E-09	2540000	Folate receptor alpha OS=Homo sapiens GN=FOLR1 PE=1 SV=3	
Q6YHK3	CD109_HUMAN	74.04	3.94E-08	6860000	CD109 antigen OS=Homo sapiens GN=CD109 PE=1 SV=2	
P29401	TKT_HUMAN	71.68	6.79E-08	2340000	Transketolase OS=Homo sapiens GN=TKT PE=1 SV=3	

Table S1. Proteomic profiles of microvesicles from PV- and mock- infected cells at 8 hpi. We report the full proteomic profile of proteins that were identified in both of two independent LC/MS experiments. Microvesicles from **PV-infected cells** carry 65 identified host protein matches (columns to the left of the green column, including Accession number, protein name, -10lgP, P, and aggregated area from one of the experiments). Microvesicles from **mock-infected cells** carry five host protein matches (columns to the right of the green column). All proteins identified in samples from mock-infected cells were also identified in samples from PV-infected cells. All proteomic analyses were conducted at a specified precursor ion (MS1) error tolerance of 10 ppm, a fragment ion (MS/MS) error tolerance of 0.02 Da. and a target-decoy false discovery threshold of 0.1%.
